# Resolving the Ortholog Conjecture: Orthologs Tend to Be Weakly, but Significantly, More Similar in Function than Paralogs

**DOI:** 10.1371/journal.pcbi.1002514

**Published:** 2012-05-17

**Authors:** Adrian M. Altenhoff, Romain A. Studer, Marc Robinson-Rechavi, Christophe Dessimoz

**Affiliations:** 1 ETH Zurich, Department of Computer Science, Zürich, Switzerland; 2 Swiss Institute of Bioinformatics, Lausanne, Switzerland; 3 Department of Ecology and Evolution, University of Lausanne, Lausanne, Switzerland; 4 Institute of Structural and Molecular Biology, Division of Biosciences, University College London, London, United Kingdom; 5 EMBL-European Bioinformatics Institute, Hinxton, Cambridge, United Kingdom; University of California Davis, United States of America

## Abstract

The function of most proteins is not determined experimentally, but is extrapolated from homologs. According to the “ortholog conjecture”, or standard model of phylogenomics, protein function changes rapidly after duplication, leading to paralogs with different functions, while orthologs retain the ancestral function. We report here that a comparison of experimentally supported functional annotations among homologs from 13 genomes mostly supports this model. We show that to analyze GO annotation effectively, several confounding factors need to be controlled: authorship bias, variation of GO term frequency among species, variation of background similarity among species pairs, and propagated annotation bias. After controlling for these biases, we observe that orthologs have generally more similar functional annotations than paralogs. This is especially strong for sub-cellular localization. We observe only a weak decrease in functional similarity with increasing sequence divergence. These findings hold over a large diversity of species; notably orthologs from model organisms such as *E. coli*, yeast or mouse have conserved function with human proteins.

## Author Summary

To infer the function of an unknown gene, possibly the most effective way is to identify a well-characterized evolutionarily related gene, and assume that they have both kept their ancestral function. If several such homologs are available, all else being equal, it has long been assumed that those that diverged by speciation (“ortholog”) are functionally closer than those that diverged by duplication (“paralogs”); thus function is more reliably inferred from the former. But despite its prevalence, this model mostly rests on first principles, as for the longest time we have not had sufficient data to test it empirically. Recently, some studies began investigating this question and have cast doubt on the validity of this model. Here, we show that by considering a wide range of organisms and data, and, crucially, by correcting for several easily overlooked biases affecting functional annotations, the standard model is corroborated by the presently available experimental data.

## Introduction

Understanding the relation between gene evolution and function is perhaps our only hope of bringing functional annotation in line with the furious pace of genomic sequencing. Indeed, despite developments in high-throughput experimental techniques, propagation of functional knowledge from evolutionarily related genes remains the procedure that scales best and appears most dependable [1]. The simplest model for this assumes that function is conserved among homologs, which motivates a process that assigns function by sequence similarity. A canonical refinement of this model distinguishes orthologs from paralogs [2], [3]. As gene duplication is considered an important source of functional innovation, the “standard model” posits that orthologs tend to have a conserved function, whereas paralogs tend to diverge in function [4].

Yet, large-scale studies corroborating this standard model are surprisingly scarce [5]. Furthermore, sequence similarity seems to be a better predictor of function conservation than orthology [6]. This suggests an alternative model, that orthologs versus paralogs might not be the primary clue to functional similarity. With the recent availability of genome-wide reliable orthology predictions on the one hand, and systematic, standardized functional annotations on the other, we now have the ability to test these models on a broad and representative sample of biological data. Recently, Nehrt et al. [7] have proposed such a test of the “ortholog conjecture” (i.e., the “standard model”), using human and mouse functional annotations. Surprisingly, they find that paralogs appear more functionally similar than orthologs.

In the present study, we investigated the functional similarity of 395,328 pairs of orthologs and paralogs with experimental GO annotations [8] for both genes, from 13 genomes (see *Materials and Methods*). After controlling for confounding factors which we describe in detail below, we find that—*contra* Nehrt et al. [7]—current experimental annotations do support the “ortholog conjecture”, albeit not as strongly as might have been expected.

## Results

### Controlling confounding factors in the comparison of GO annotations

**Figure 1:**
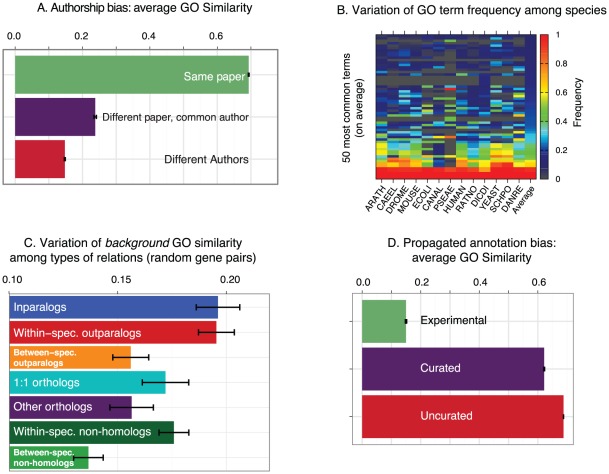
Potential confounding factors in GO analyses. (**A**) Authorship bias: average GO Similarity of homologs pairs partitioned according to their provenance. (**B**) Variation of frequencies of GO terms among the 13 analyzed genomes (50 most common terms on average depicted). (**C**) Average background frequency for the different subtypes of gene pairs, obtained by computing the average similarity of random pairs from sequences involved in the respective categories. (**D**) Average GO similarity between homologous gene pairs partitioned according to their GO annotation evidence tags (*Experimental*: evidence code EXP and children; *Uncurated*: evidence code IEA; *Curated*: all other evidence codes). To compute the average similarity for each category, annotations from the other 2 categories are filtered out.

GO annotations—even restricting to experimentally supported ones—are heterogeneous in many ways, such as type of function described, level of specificity, applicable species, method of investigation, or curation practices [9]. Therefore, to meaningfully compare GO annotations, it is essential that potential confounding factors be controlled. In this section, we describe and address four confounding factors (Fig. 1): (i) authorship bias, (ii) variation of GO term frequency among species, (iii) variation of background similarity among species pairs, and (iv) propagated annotation bias. To our knowledge, the effect of these factors has not been clearly reported previously.

#### Authorship bias

The average GO similarity of genes annotated based on the same scientific article is higher than that of genes annotated based on different articles (Fig. 1A; Mann-Whitney U test, p<2.2 10^−16^) [7]. Furthermore, even for annotations derived from different articles, the average GO similarity of homologous genes is significantly higher when these articles have at least one author in common (Fig. 1A; Mann-Whitney U test, p<2.2 10^−16^), an effect never reported previously to our knowledge. These effects are highly relevant to the present analysis, because the distribution of orthologous and paralogous pairs among these 3 categories is extremely skewed (Fig. S1): the function of within-species paralogs is ∼40 times more frequently annotated from the same article than that of orthologs (Table S1, S2). Presumably, genes within the same species tend to be studied by the same investigators, based on similar experiments, and using similar terminology to describe their results. To control for authorship bias, we restrict all analyses below to annotations derived from distinct articles sharing no common author.

#### Variation of GO term frequency among species

Typical measures of function similarity do not account for variation of GO term frequency among species. This is the case for measures defined on the ontology graph alone, such as term overlap measures (e.g., Jaccard index [10] or Maryland-bridge coefficient [11]). But even measures based on information content usually rely on GO term frequencies estimated from the entire database [12], thereby implicitly assuming that the frequency of GO terms is uniform across all species. However, the frequency of GO terms varies considerably across species (Fig. 1B). Thus, to take this into account, we estimate the frequencies of GO terms separately for each species (see *Materials and Methods*).

#### Variation of background similarity among species pairs

Even if we account for variation in GO term frequency among species, the average similarity of random pairs of genes (which we call “background similarity”) is not equal for all genome pairs (Fig. S2). Indeed, the background similarity depends on other factors which vary among genomes, such as the degree of annotation coverage (i.e., the average number of GO term per gene). Crucially in the context of the Biological Process ontology, the background similarity for genes within the same genome tends to be highest (Fig. S2). Thus, background similarity is much stronger for paralogs (which can be within the same species) than for orthologs (by definition in different species) (Fig. 1C). To avoid this problem, we normalize all measures of function similarity with respect to background similarity, and for subsets of homologs detected between genomes (see *Materials and Methods*).

#### Propagated annotation bias

Experimentally backed GO annotations (evidence code EXP and children), which constitute less than 1% of all annotations, are undisputedly considered the most reliable [12]. The rest of the annotations are mainly inferred by function propagation among homologous sequences, which are detected by sequence similarity. Even when propagation takes place through manual curator intervention, this process introduces a bias in the distribution of annotations. Indeed, the average function similarity of homologs as a function of sequence divergence is very different for experimental, curated (non-experimental), and automated annotations (Fig. 1D; Kruskal-Wallis test, p<2.2 10^−16^). The most probable interpretation is that since propagated annotations are inherently identical to their source, extensive term propagation inflates the average GO similarity of homologs, especially with similar sequences. As we show below, a similar trend is observable with Enyzme Commission (EC) number annotations.

### Yeast-only comparison

Correcting for the biases described above, we first restricted our comparison to experimental annotations with no common investigator from the two yeast species, *Saccharomyces cerevisiae* and *Schizosaccharomyces pombe*. They were chosen because (i) they form the pair of species with the most ortholog pairs which both have experimentally supported GO annotations; (ii) they are quite similar in biology, and are studied by scientific communities with similar interests; and (iii) since horizontal gene transfer is relatively rare in eukaryotes, the distinction between orthology and paralogy is conceptually straightforward. Thus, we hope to minimize organism specific annotation biases. Function similarity was computed using an information-theoretic measure taking into account the variation of annotation coverage among species (Fig. 1B), and normalizing with respect to the background similarity of random gene pairs (Fig. 1C; for details, see *Materials and Methods*).

**Figure 2:**
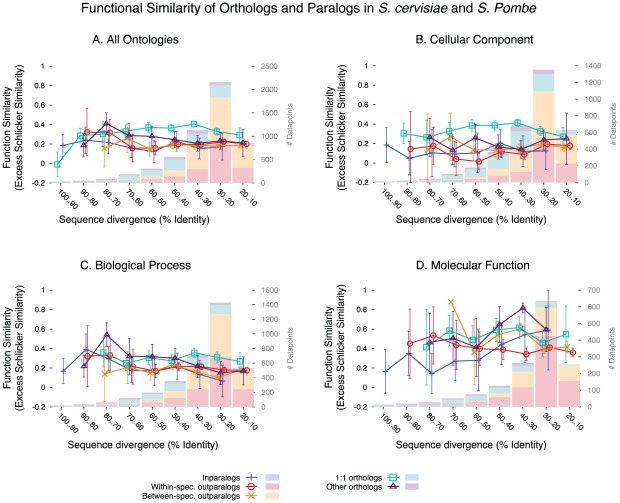
Function similarity of the different types of homologs, in yeasts. Only pairs of annotations derived from different publications, which do not share any common author, were used. (**A**) over all Gene Ontology annotations; (**B**) restricted to the Cellular Component ontology; (**C**) restricted to the Biological Process ontology; (**D**) restricted to the Molecular Function ontology. Histograms represent sample density partitioned for each homology type, and error bars represent the 95% confidence interval around the mean.

The first observation is that at similar levels of sequence divergence, one-to-one orthologs do have significantly more similar experimental GO annotations than paralogs, and that one-to-many and many-to-many orthologs (referred to as “other orthologs” in the remainder of the text) are somewhat intermediary (Fig. 2A) (Kruskal-Wallis test between homology types, *p*<2.2 10^−16^; t-test of 1∶1 orthologs *vs.* other homologs, *p*<2.2 10^−16^); this is consistent with the ortholog conjecture. The difference of excess similarity between one-to-one orthologs and other homologs is considerable (average functional similarity of 0.36 vs. 0.20). Also consistent with expectations, there is almost no difference between same-species paralogs and different-species paralogs (t-test, *p* = 0.029; difference of 8%). It is difficult to tell whether this small difference is biologically relevant, or whether it corresponds to some residual species-specific annotation bias.

On the other hand, the difference between orthologs and paralogs is not as important as might have been expected under a naive interpretation of the ortholog conjecture: orthologs are far from having almost the same function. This might stem in part from the differences between experiments performed by different investigators. Most surprising, the decrease in annotation similarity with protein divergence is very weak (Spearman correlation between sequence identity and GO similarity over all homologs: ρ = −0.019, *p* = 0.009). This contradicts the predominant notion that “sequence divergence is generally accompanied by higher likelihood of divergence in function” [13].

We have verified these results with a number of additional controls: using different metrics of GO annotation similarity (Fig. S3); using different metrics of protein divergence (Fig. S4); and using only gene quartets with orthologs and paralogs in both species (Fig. S5). In all cases, we recover the higher functional similarity of orthologs than paralogs, and the low correlation between annotation similarity and protein divergence. Furthermore, to assess the significance of the difference between orthologs and paralogs for each level of sequence divergence, we performed bin-by-bin non-parametric Mann-Whitney U tests (Fig. S6). All tests that are significant at the 99% confidence level showed an excess of similarity of orthologs over paralogs (Table S3).

The GO is composed of three orthogonal ontologies, which we have analyzed separately for the two yeasts. The Cellular Component ontology shows the most marked pattern, with a very clear excess of similarity between one-to-one orthologs, relative to all other homologs (Fig. 2B; t-test, *p*<2.2 10^−16^; difference of 57%). Orthologs are also very significantly more similar for Biological Process (Fig. 2C; t-test, *p*<2.2 10^−16^; difference of 41%), whereas for Molecular Function the difference is weaker (Fig. 2D; t-test, *p* = 1.6 10^−7^; difference of 30%). The difference is a bit stronger for Molecular Function if all orthologs are contrasted to all paralogs (t-test, *p* = 3.9 10^−12^), but it remains weaker than for the other two ontologies.

One inherent limitation of two-species analyses is that all pairs of orthologs started diverging at the same time (the speciation event between the two species), with almost all paralogs being either older (the “out-paralogs”) or younger (the “in-paralogs”) than the orthologs. By considering sequences from many different gene families—some of which faster evolving, other slower evolving—we can compare orthologs and paralogs that have similar levels of sequence divergence, but inevitably, slow-evolving orthologs will tend to be compared with in-paralogs, while fast-evolving orthologs will tend to be compared with out-paralogs. To avoid the potential bias that this might introduce, we need to look at data from multiple species.

### All-against-all species comparisons

**Figure 3:**
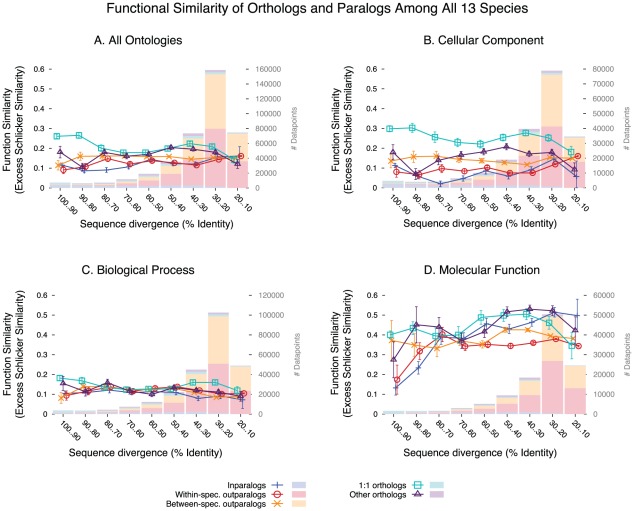
Function similarity of the different types of homologs, for all 13 genomes. Only pairs of annotations derived from different publications, which do not share any common author, were used. (**A**) over all Gene Ontology annotations; (**B**) restricted to the Cellular Component ontology; (**C**) restricted to the Biological Process ontology; (**D**) restricted to the Molecular Function ontology. Histograms represent sample density partitioned for each homology type, and error bars represent the 95% confidence interval around the mean.

We performed the same comparisons between all possible pairs of the 13 species with sufficient experimental GO annotations. Results are widely consistent with the yeast only study (Fig. 3; Fig. S7): at similar levels of sequence divergence, orthologs, and especially one-to-one orthologs, are more similar in GO annotations than paralogs, although the absolute difference is modest. Likewise, the difference between same-species paralogs and different-species paralogs is still quite modest (t-test, *p*<2.2 10^−12^; difference of 10%). We also confirm that the excess similarity of orthologs vs. paralogs is strongest for the Cellular Component ontology. With this larger and more diverse dataset, the excess similarity of orthologs is also highly significant for the Molecular Function ontologies (all orthologs vs. all paralogs, t-test, *p*<2.2 10^−16^), as for the Biological Process (Fig. 3). To assess the significance of the difference between orthologs and paralogs for each level of sequence divergence, we also performed bin-by-bin non-parametric Mann-Whitney U tests (Fig. S8; Table S3). They were significant and consistent with the general trend of orthologs more functionally similar than paralogs for all but very divergent sequences (10–20% range of sequence identity), where there is a slight excess of similarity for paralogs (difference of 0.0250, p-value = 0.00022). But it should be noted that 10–20% identity is well into the twilight/midnight zone, where even homology calling is difficult, let alone orthology/paralogy calling. As additional controls, we confirmed that our results are not sensitive to the choice of bin size (Fig. S9), function similarity measure (Fig. S10), or overrepresented gene families (Fig. S11, 12). Furthermore, the results are also supported by analyses performed on Ensembl compara data, an alternative source of orthologs/paralogs sequence pairs ([14]; Fig. S13).

**Figure 4:**
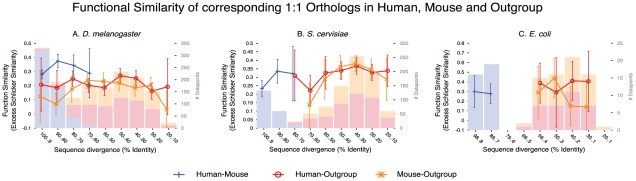
Function similarity (all ontologies combined) of corresponding 1∶1 orthologs among human, mouse, and an outgroup. The outgroups are (**A**) *D. melanogaster*, (**B**) *S. cervisiae*, and (**C**) *E. coli*. Strikingly, function is maintained among 1∶1 orthologs over all evolutionary ranges considered here. Histograms represent sample density partitioned for each homology type, and error bars represent the 95% confidence interval around the mean.

Like for the yeast study, there is little correlation between functional similarity and protein sequence identity (Spearman ρ = −0.023, *p*<2.2 10^−16^). The correlation with species divergence time is also very weak (Spearman ρ = −0.052, *p*<2.2 10^−16^ 10^−12^; computed only on orthologs; Fig. S14c). A potential confounding effect is that only well-conserved proteins can be detected as homologs between distantly related organisms. To control for this effect, we compared annotations of orthologs conserved among triplets of genomes (Fig. 4). For the human-mouse-fly triplet, functional similarity is stronger between human and mouse than with fly. But for triplets involving yeast or *E. coli*, functional similarity is the same between human or mouse and the third genome, as between human and mouse. Of note, the GO similarity of human or of mouse to the outgroup is always extremely similar, despite using independently generated annotations.

### Enzyme commission numbers

Enzyme commission (EC) numbers are an alternative source of functional annotations. The relation between EC numbers and sequence divergence has already been studied extensively (e.g., [15]), especially before GO supplanted EC as the main source of functional annotations, but is restricted to genes with catalytic activity. In relative terms, the functional similarity of orthologs and paralogs in terms of EC numbers behaved like experimental GO annotations, with 1∶1 orthologs showing the highest level of similarity, other orthologs a somewhat lower level, and paralogs the lowest level (Fig. S13). In absolute terms, however, average functional similarity for all categories was generally higher and decreased much more distinctly with decreasing percentage identity (Spearman ρ = 0.45 p<10^−16^). As we note above, computational propagation of functional annotations inflates functional similarity in absolute terms. Since there is no evidence code used for EC annotations, most of the comparison is based on computational propagation. This could also explain the stronger decrease, as propagation preferentially takes place between homologs close in sequence.

## Discussion

The distinction between orthologs and paralogs has been a central concept of phylogenomics [3]. And yet, it is only recently that the functional relevance of this distinction has been treated as a hypothesis to be tested. To date, several indirect, sequence-based studies have failed to support this classical model, rather supporting an alternative model of uniform functional divergence, independent of duplication [reviewed in 5]. Recently, Nehrt et al. [7] have compared the functional annotations of orthologs and paralogs between human and mouse. Surprisingly, they report the strongest functional similarity for paralogs, which is expected neither under the classical model nor under the uniform model.

Directly comparing functional annotations is complicated, because they are derived from a variety of sources and by a variety of procedures. The best-known bias is that computationally derived annotations (IEA code) are generally believed to be less reliable than experimentally derived annotations. The computational annotations reflect the algorithms used to propagate annotations [16], and thus are shared preferentially among proteins with high sequence similarity, among orthologs, or among proteins sharing well-defined domains. Any analysis including these GO annotation will recover the impact of these algorithms, which is indeed what we find when we use all GO annotations. Much of the older literature on function divergence used the EC nomenclature as a measure of function, and thus mixed indiscernibly electronic and experimental annotations. Thus it is probable that most results based on the EC nomenclature are biased by electronic annotations (i.e., Fig. S15).

Even limiting ourselves to experimentally derived annotations, there remains a great deal of complexity and bias in the data of functional annotation.

First, different model organisms are studied by different scientific communities, for different purposes, which bias the types of experiments conducted and reported. Moreover, each organism is predominantly annotated by one Model Organism Database team, which differs from others in its data curation and annotation practices. Indeed, we observe significant differences in background functional similarity, depending on the species compared. While part of this variation might be due to biological differences among the species, these differences appear to be mostly due to the artifacts outlined above. Here, we have compared 13 organisms spanning the tree of life (Fig. S17; Table S4), and we have corrected each comparison by the background frequencies of annotations from the relevant genomes. Moreover, we show that results limited to two yeasts are consistent with results averaged over all organisms.

Second, each experiment is performed and reported by a given team of investigators, who have a scientific focus and a manner of reporting which are specific to them. This induces a strong bias towards similar annotations derived from the same paper, which mostly affects same-species paralogs. Importantly, there is a bias towards similar annotations even when considering different papers which share at least one co-author. Unless accounted for, this confounding factor leads to a large spurious excess of similarity between same-species paralogs [similar to the results of 7]. Controlling for it leads to the opposite conclusion: a weak excess of similarity between orthologs (Fig. S16). This observation is also corroborated by a recent rebuttal from the GO consortium, which reexamined two case studies from Nehrt et al.'s paper and concluded that the difference in function similarity computed between orthologs and paralogs was mainly due to bias in annotations, not in the underlying functions [9].

While GO annotations are complex and biased, it nevertheless appears possible to identify and correct these biases, and to detect biologically significant signal. We feel that the use of 13 different species, with diverse annotation levels and evolutionary distances, contributes to the robustness of our results.

Once the biases identified above are accounted for, the signal which emerges can be summarized in three major points: (i) Consistent with the “ortholog conjecture”, or “standard model of phylogenomics”, overall functional similarity is highest between one-to-one orthologs, lowest between paralogs, and intermediate between other orthologs. (ii) There is at best a very weak relation between protein sequence similarity and functional similarity. (iii) The difference between orthologs and paralogs, although consistent with the ortholog conjecture, is weaker than expected under a naive understanding of that model; this is especially true when Molecular Function and Biological Process are considered separately.

The standard model of higher functional similarity among orthologs than paralogs at similar levels of sequence divergence could not be supported until it was explicitly tested [5]. Several recent studies have performed such tests, and found some measure of support for the standard model. On a structural level, there appears to be higher conservation of intron position [17], of protein structure [18], and of domain architecture [19] between orthologs. Presumably more relevant to biological function, the conservation of expression patterns appears higher between orthologs than between paralogs, in mammals [20]. On the other hand, Nehrt et al. [7] have found that the expression correlation of human/mouse inparalogs is significantly higher than that of orthologs (but not outparalogs). And a study of the evolution of sub-cellular localization in yeasts did not find any difference between orthologs and paralogs [21]. These contradictory results might be due in part to the overall modest difference between orthologs and paralogs, and in part to differences between different aspects of function.

An intriguing pattern in our results is that we find strong conservation of Cellular Component annotations among orthologs. Contrary to the two other ontologies, sub-cellular localization is an aspect of function which leaves little room for divergent interpretation. Moreover, experimental results are easier to report in similar terms in different species. These factors might allow better detection of the excess conservation of orthologs. Thus, of the 3 ontologies, our results on cellular components are arguably the most conclusive.

As for the two other aspects of protein function captured by the Gene Ontology—Molecular Function and Biological Process—they have more subtle patterns. Molecular Function shows an excess of conservation between orthologs which is weaker than for Cellular Component, but which is strongly significant over all 13 genomes analyzed. This is the aspect of function for which there was previously the most evidence for the “uniform model” of no significant difference between orthologs and paralogs; with the available data, this can now be rejected. This is also the aspect of function for which the absolute value of excess similarity (i.e., excess similarity of homologs over random pairs) is strongest—for both orthologs and paralogs. Thus, Molecular Function appears to be strongly conserved between even distant homologs, which supports the received wisdom of predicting this type of annotation on the basis of conserved protein domains.

Biological Process also has a significant excess of function conservation among orthologs, although weaker than for the Cellular Component. This is surprising, given the wide differences in biology between the species compared. Indeed, throughout the entire range of sequence divergence, orthologs are considerably more similar in function than even same-species paralogs. Of note, the biases which amplify apparent similarity between paralogs are strongest for this aspect of function: not correcting for the sampling bias of orthologs or paralogs detected between species can lead to a spurious excess of conservation of same-species paralogs. Our results contradict the concept of the evolution of cellular context set forth by Nehrt et al. to explain the apparent higher similarity of function of in-paralogs between human and mouse [7].

This concept was also related to the weak relation between protein sequence divergence and functional divergence. Nehrt et al. [7] speculated that protein function might evolve more as a function of the divergence of cellular context than as a function of protein sequence. They suggested that a comparison of orthologs of different ages might recover an effect of divergence age on functional divergence. Our analysis includes species divergences spanning the range from 36 Mya to 3300 Mya, yet we still do not find a strong relation between functional divergence and protein divergence, nor with species divergence time. These observations suggest that protein function evolves in a very non-clock-like manner. Indeed, clock-like evolution is an expected pattern for neutrally evolving characters [22], whereas selection is expected to be the major force shaping the evolution of protein function.

The low impact of evolutionary time on average protein function conservation is also apparent if we compare humans to model organisms with very different divergence times. Indeed, the extent of functional similarity of one-to-one orthologs is similar between human and *E. coli*, human and yeast, human and fly, or human and mouse. This supports the strong relevance of these various species for understanding human biology. In fact, the average similarity over all available one-to-one orthologs is even higher for the more distant *E. coli* and yeast, than for fly or mouse. This is probably due the fact that only proteins with very strong function conservation are kept as detectable one-to-one orthologs over such long evolutionary spans. We verified this by comparing only proteins which are detected as one-to-one orthologs in triplets of these species. For human-mouse-fly, we do recover a stronger similarity for more closely related species. But for the triplets with yeast or *E. coli*, this is not the case. In terms of evolutionary biology, this shows that, to some extent, protein function does diverge with time. Yet there is a class of proteins, conserved beyond animals, which conserve their function, irrespective of divergence time, on average. In terms of annotation procedures for databases, and even design of new experiments, these results show that if a protein is conserved between two species, as one-to-one ortholog, then its function is probably mostly conserved, even if the divergence time is very large.

In conclusion, our analyses corroborate the central tenet of the standard model of phylogenomics—that at similar levels of sequence divergence, orthologs are in general more similar in function than paralogs. But although significant, the difference is modest, and is uneven among different aspect of function (among different ontologies). Furthermore, our results expose other trends unexplained by the standard model, such as differences among subtypes of orthology and paralogy (also observed in other contexts, such as intron conservation [17]), or the lack of interaction between sequence and function divergence. Hence, the standard model has validity, but is of only limited practical use. To further progress in our understanding of the relation between gene evolution and gene function, we need to move beyond the orthology/paralogy dichotomy.

## Materials and Methods

### Data collection

We selected 13 genomes with highest coverage in GO annotations backed by experimental data (evidence codes EXP, IDA, IEP, IGI, IMP, and IPI). The annotations were retrieved from the GOA database [16] release 73 and Ensembl [23] release 54. We used orthologs and paralogs inferred by OMA [24], [25]. For each species pair, we extracted all the one-to-one orthologs, all other orthologs (one-to-many and many-to-many), all out-paralogs (within and between the species pair) and all inparalogs (in this context by definition only within the same species). Only gene pairs with more than 10% identity in amino-acid sequence were kept. This yielded a total of 9,564,666 pairs of genes. Of those, 395,328 had experimental GO annotations for both genes. We computed the similarity of these experimental annotations using several measures (see below), with evolutionary distance in percent sequence identity computed over the total protein sequences as independent variable.

### Alternative functional annotations

We used the EC number assignments of the ENZYME database, maintained by Swiss-Prot [26].

### Alternative orthology/paralogy source

We used orthologs and paralogs induced by Ensembl Compara gene trees (version 65) [23] together with GO annotations from GOA (release 2012-01-21) as an alternative dataset (Fig. S13).

### Measures of functional similarity

The comparison of gene annotations requires a measure of semantic similarity. In recent years, several measures have been proposed (for review, [27]). In the present context, 3 aspects of these metrics are most relevant: (i) how to compute the similarity between two GO terms, (ii) how to deal with multiple terms for a given gene, and (iii) how to normalize the measure across species.

#### Similarity measure between two terms

A first similarity measure is Resnik's information content metric [28], which is computed from the probability of the most specific term that subsumes the two annotations in question *c_i_, c_j_*:
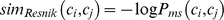



This measure is directly related to the information content of the most specific common parent of the two terms. The higher this value, the more specific the communality of the annotations. Note that the probabilities for all terms are commonly estimated from their frequency of occurrence in the database. A natural extension is Lin's [29] metric, which normalizes Resnik's measure by the average information contained in the two annotations themselves:
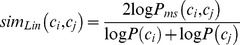



Therefore, Lin's similarity is bounded between 0 (related only through the root ontology term) and 1 (identical annotations).

#### Similarity measure between two genes

Genes are often annotated with more than one term, which raises the question of how to compute the overall similarity between two genes. Two common approaches consist in computing the similarity for all pairs of GO terms between the two genes, and to report either the maximum or the average among them. To overcome problems with these measures [12], Schlicker *et al* [30] have suggested to average only over the most similar counterparts of each term. Based on this idea, we use the following similarity measure:
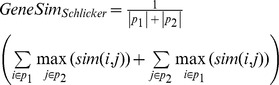
where *p*_i_ is the set of GO terms associated with protein i, and 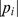 its cardinality. Note that this formulation differs from Schlicker et al's in the way the maxima terms are averaged: we give each *annotation* the same weight, while they give *each gene product* the same weight. Unless noted otherwise, we use our variant of Schlicker's averaging of Lin's GO term similarity.

As alternative to these information-theoretic motivated similarity measures, similarity measures based solely on the ontology graph (e.g. term-overlap measures) have also been proposed and applied e.g. Jaccard index [10] or Maryland-bridge coefficient [11].

In order to compare our results with the findings of Nehrt et. al [7], we also analyzed our data using the Maryland-bridge measure:
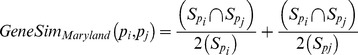
where S_p_ is the set of GO annotation term p and all their propagated parent terms (except for the ontology root terms).

#### Normalization

As not all genomes are annotated by the same people for the same purpose, there can be substantial differences in annotation structure and frequency across genomes. We normalize the similarity measures in two respects. First, contrary to the common practice of computing frequencies for each GO term across the entire annotation corpus [27], we estimated distinct GO term probabilities for each genome. That way, we could account for varying GO term frequencies across genomes. Thus, Lin's similarity between terms *c_i_ and c_j_* becomes dependent on the genomes g_I_, g_I_ in which they occur:
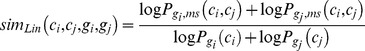
where 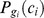 is the frequency of term *c_i_* in genome g_i_, and 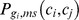 the frequency of the most specific common parent of *c_i_* and *c_j_* in genome g_i_.

The second normalization step is motivated by the observation that the average similarity of random pairs of genes (the background similarity) is not equal for all genome pairs and subtypes of homology (Fig. 1c; Fig. S2). For instance, the background similarity is influenced by the degree of annotation coverage of a genome (i.e. the average number of GO term per gene). Also, single-copy, universal genes often have different background distribution among functional categories than their multi-copy counterparts. Thus, for all pairs of genomes (including self-pairs) and every type of homologous relation, we estimated the background by computing the average similarity of 10,000 random pairs of annotated genes, sampled with replacement. The normalized measure, which we call *excess similarity*, is thus defined as
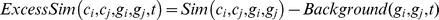
where c_i_, c_j_ are the terms, g_i_, g_j_ the genomes, and t the homology subtype.

### Authorship bias

For each GO annotation an evidence code and a reference identifier is recorded. In the case of experimental annotations (EXP, IDA, IEP, IGI, IMP and IPI), this reference id is usually a PubMed identifier or a reference id from a model organism database (MOD). We extract authors associated with a given GO annotations by first mapping non-PubMed reference ids to PubMed ids using publicly available mapping files from the MODs. Second, for each PubMed id we extract the authors of that publication from the PubMed webpage.

## Supporting Information

Dataset S1 Tab-separated text file with all raw data of the main dataset (experimentally-supported GO annotations without common authors).
(GZ) Click here for additional data file.

Figure S1 Contrasting excess Schlicker-like similarity of homologs with experimental annotations reported in A) the same publication, B) different publications involving at least one common author and C) publications with different authors only.
(PDF)

Figure S2 Estimated background similarity per genome pair for each ontology and homolog relation type. For within-species homologs, entries along one column correspond to the background similarity within the species on the x-axis with respect to the speciation event with the species on the y-axis. The background similarities for each genome pair and homology type have been computed between 10,000 random gene pairs, where both genes have (i) at least one recorded homologous match of that type and (ii) are annotated with experimental GO annotations.
(PDF)

Figure S3 Different measures of GO term similarity among various types of homologs. The six figures are A) maximum sim_Resnik_, B) average sim_Resnik_, C) maximum sim_Lin_ and D) average sim_Lin_, E) Maryland-bridge term overlap measure, F) sim_Schlicker_ (giving same weight to annotation) and G) sim_Schlicker_ as originally defined in Schlicker et. al (2006) (giving same weight to each gene product). All similarities are measured from the YEAST/SCHPO comparison with GO annotations backed by experimental evidence without common authors.
(PDF)

Figure S4 Contrasting different measures of divergence as independent variables: A) Percent sequence identity and B) PAM estimates of sequence divergence, both derived from a Smith-Waterman alignment over the full protein lengths. All function similarities are in Excess Schlicker-like Similarity and have been measured from the dataset with only GO annotations backed by experimental evidence originating from publications sharing no common authors.
(PDF)

Figure S5 Average excess Schlicker-like Similarity measured from homologous gene pairs with GO annotations backed by experimental evidence from publications with no common authors. The sampled gene pairs form quartets with an ancient duplication and subsequent speciations. The quartets are sampled from A) the two yeast species only and B) from all 13 analyzed species.
(PDF)

Figure S6 Difference in average Excess Schlicker Function Similarity between all types of Orthologs and all types of Paralogs from the YEAST/SCHPO genome pair on the dataset of pairs being backed with experimental annotations from studies without common authors. The different panels report the difference for the different GO ontologies. The data-points indicate the difference of the means and the gray area a linear interpolation of the bin-wise 95% confidence interval for the difference for the mean. To confidence interval is computed for each bin with a Mann-Whitney test. P-values are provided in Table S4 for all bins.
(PDF)

Figure S7 Average excess Schlicker-like similarity for any pair of analyzed species, measured on the dataset restricted to experimental annotations from publications without common authors. Reported is the average excess similarity over all three GO ontologies. A mapping of the species abbreviations to scientific names is provided in Table S3.
(PDF)

Figure S8 Difference in average Excess Schlicker Function Similarity between all types of Orthologs and all types of Paralogs from all 13 analyzed genomes on the dataset of pairs being backed with experimental annotations from studies without common authors. The different panels report the difference for the different GO ontologies. The data-points indicate the difference of the means and the gray area a linear interpolation of the bin-wise 95% confidence interval for the difference for the mean. To confidence interval is computed for each bin with a Mann-Whitney test. P-values for the statistical test whether the difference is different from 0 are available in Table S4 for each distance bin.
(PDF)

Figure S9 Different bin-widths (columns) for evolutionary divergence categories: the results are robust with respect to the choice of bin width. The analysis is done on the gene pairs with experimental GO annotations without common author between all 13 genomes.
(PDF)

Figure S10 Different measures of GO term similarity among various types of homologs. The six figures are A) maximum simResnik, B) average simResnik, C) maximum simLin and D) average simLin, E) Maryland-bridge term overlap measure, F) simSchlicker (giving same weight to annotation) and G) simSchlicker as originally defined in Schlicker et. al (2006) (giving same weight to each gene product). All similarities are measured from the gene pair s from all 13 analyzed genomes with GO annotations backed by experimental evidence without common authors.
(PDF)

Figure S11 Test of over-representation of a single species pair. We applied the following re-sampling strategy to the dataset of gene pairs with experimental GO annotations without common authors: First, we partition the dataset into independent sub-datasets. Each sub-dataset is composed of all the gene pairs of a given homology type and species pair. After building those sub-datasets, we randomly select gene pairs with replacement of the same size or a maximum number of allowed pairs. This number has been set to 2000 gene pairs per species pair and homology type. This way we ensure that any species pair can influence the results more than 1.5%. We then compute the average similarity per homology type and distance category from the combined sub-datasets. This whole procedure is repeated 100 times in order to obtain the necessary quantiles for the box-plots.
(PDF)

Figure S12 Test for over-representation of large gene families in the OMA homologs. We applied the following re-sampling strategy to the dataset of gene pairs with experimental GO annotations without common authors: First, we partition the dataset into independent sub-datasets. Each sub-dataset is composed of all the gene pairs from a given gene family. After building those sub-datasets, we randomly select gene pairs with replacement of the same size or a maximum number of allowed pairs. This number has been set to 100 gene pairs per gene family. This way we ensure that any single family can influence the results more than 1%. We then compute the average similarity per homology type and distance category from the combined sub-datasets. This whole procedure is repeated 100 times in order to obtain the necessary quantiles for the box-plots. For every gene family, we sample at most 100 homologous gene pairs with replacement. Shown are box-plots for all 100 bootstrap samples.
(PDF)

Figure S13 Orthology/Paralogy relations inferred from Ensembl Gene Trees (version 65). To control for a potential bias in the orthology/paralogy inference method we repeated the analysis on homologs induced by the labeled Ensembl gene trees. Note that this analysis is limited to the following 6 species: HUMAN, MOUSE, RATNO, DROME, CAEEL and YEAST. Shown are the excess Schlicker similarities. In all ontologies, orthologs are significantly more similar in function than paralogs. The figures show the similarities of A) the average over all gene ontologies (t-test: p<2.2E−16), B) the molecular function ontology (t-test: p<2.2E−16), C) the biological process ontology (t-test: p = 2.19E−6) and D) the cellular component ontology (t-test: p<2.2E−16). All similarities have been computed on the dataset with experimental annotations without common authors from GOA 2012-01-21.
(PDF)

Figure S14 Contrasting different measures of divergence as independent variables: A) Percent sequence identity, B) PAM estimates of sequence divergence and C) Time estimates. Time estimates have been extracted from TimeTree (http://timetree.org). All function similarities are in Excess Schlicker-like Similarity and have been measured from the dataset with only GO annotations backed by experimental evidence originating from publications sharing no common authors.
(PDF)

Figure S15 Average excess Schlicker-like similarity of the various types of homologs with EC number annotations, with sequence divergence in percent identity as independent variable.
(PDF)

Figure S16 Effect sequence on functional similarity after correcting for several biases for A) biological process, B) cellular component and C) molecular function GO ontology. Homologs are taken from Nehrt *et. al* (2011), and initial plots are computed on experimental GO annotations augmented with curated annotations having *TAS* or *IC* evidence code. In the subsequent plots, we correct for author bias (only annotations from publications without common author), curator effect (by only looking at experimental annotations), varying background and information content based similarity measure.
(PDF)

Figure S17 The 13 species used in the analysis and their phylogenetic relations among each other according to the NCBI taxonomy.
(PDF)

Table S1 Authorship bias: the fraction of homologs with experimental GO annotations from the same publication, different publication but common author and different authors varies strongly. All homologs have at least 50% sequence identity.
(PDF)

Table S2 Authorship bias: equivalent to Table S1, but without restriction on the sequence conservation.
(PDF)

Table S3 Significance test for difference of mean excess Schlicker-like similarity between orthologs and paralogs. P-values have been computed for each distance bin separately using a Mann-Whitney test. Values are shown for the dataset covering all 13 genomes (middle column) as well as the yeast-only dataset (rightmost column). The corresponding graphs are provided in Fig. S6 (yeast only) and S8 (all species).
(PDF)

Table S4 Species information: source and release date for all 13 analyzed species. Their phylogenetic relation is depicted in Fig. S17.
(PDF)
