# Supplementary material for: Resolving the Ortholog Conjecture: Orthologs Tend to Be Weakly, but Significantly, More Similar in Function than Paralogs
Source: PLoS Comput Biol. 2012 May 17;8(5):e1002514. doi: 10.1371/journal.pcbi.1002514 (PMC3355068; doi:10.1371/journal.pcbi.1002514)

Authorship Bias

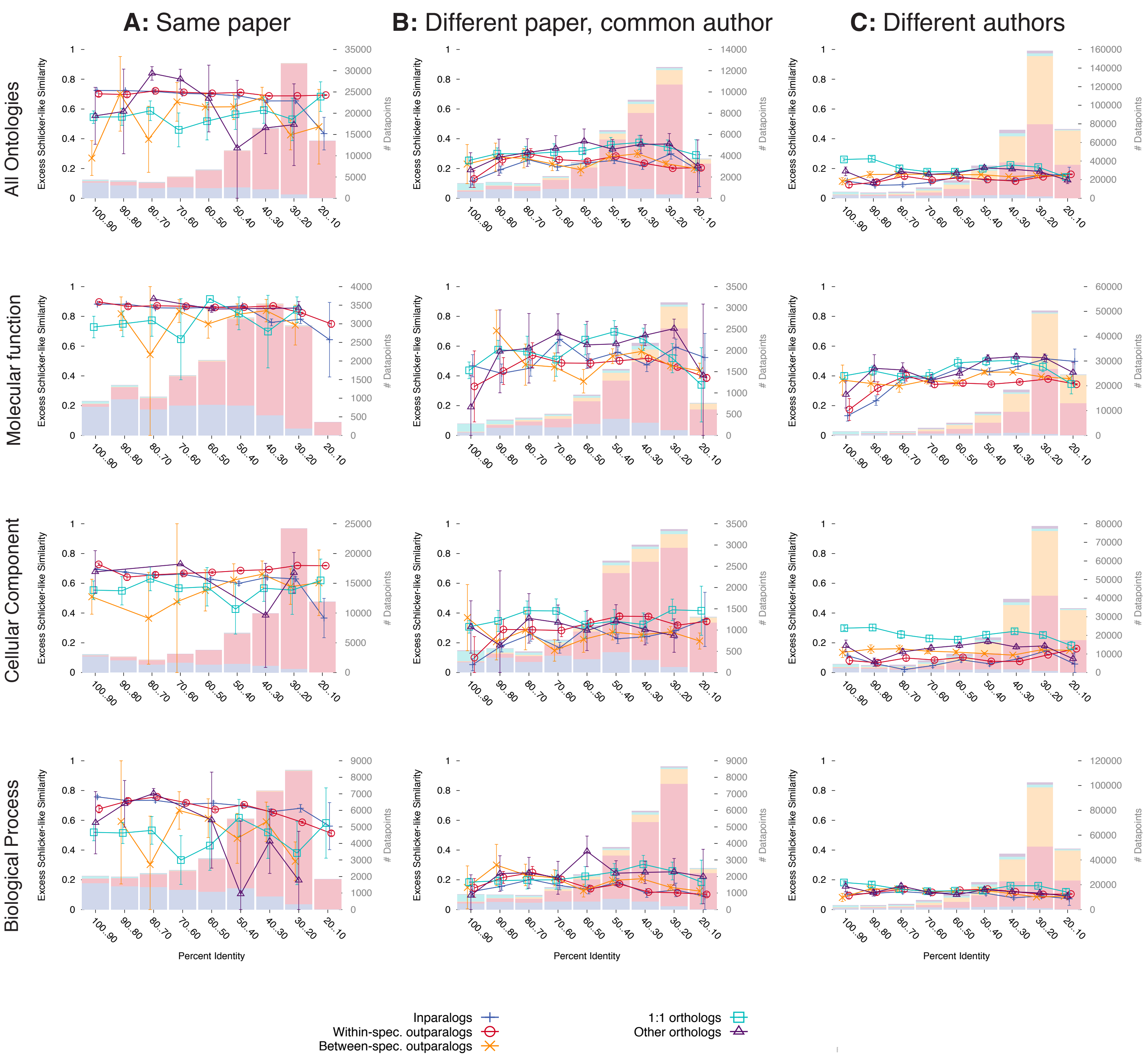

Supplement: Figure S1 — Contrasting excess Schlicker-like similarity of homologs with experimental annotations reported in A) the same publication, B) different publications involving at least one common author and C) publications with different authors only. (PDF) [file pcbi.1002514.s002.pdf]
