# Supplementary figures and images for: Resolving the Ortholog Conjecture: Orthologs Tend to Be Weakly, but Significantly, More Similar in Function than Paralogs
Source: PLoS Comput Biol. 2012 May 17;8(5):e1002514. doi: 10.1371/journal.pcbi.1002514 (PMC3355068; doi:10.1371/journal.pcbi.1002514)

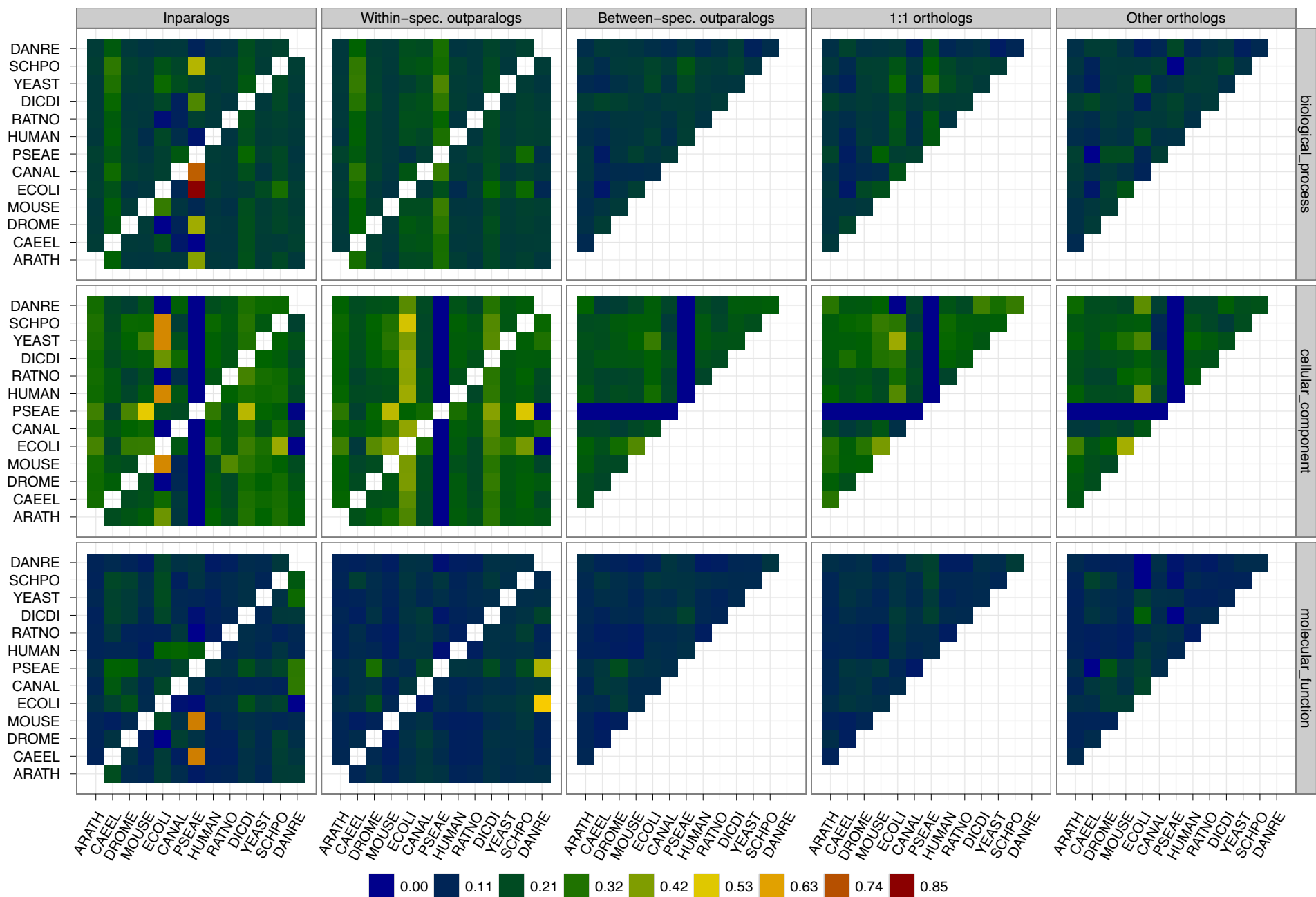

Supplement: Figure S2 — Estimated background similarity per genome pair for each ontology and homolog relation type. For within-species homologs, entries along one column correspond to the background similarity within the species on the x-axis with respect to the speciation event with the species on the y-axis. The background similarities for each genome pair and homology type have been computed between 10,000 random gene pairs, where both genes have (i) at least one recorded homologous match of that type and (ii) are annotated with experimental GO annotations. (PDF) [file pcbi.1002514.s003.pdf]

# Different metrics of protein divergence (Yeasts only)

## A: Percent Identity

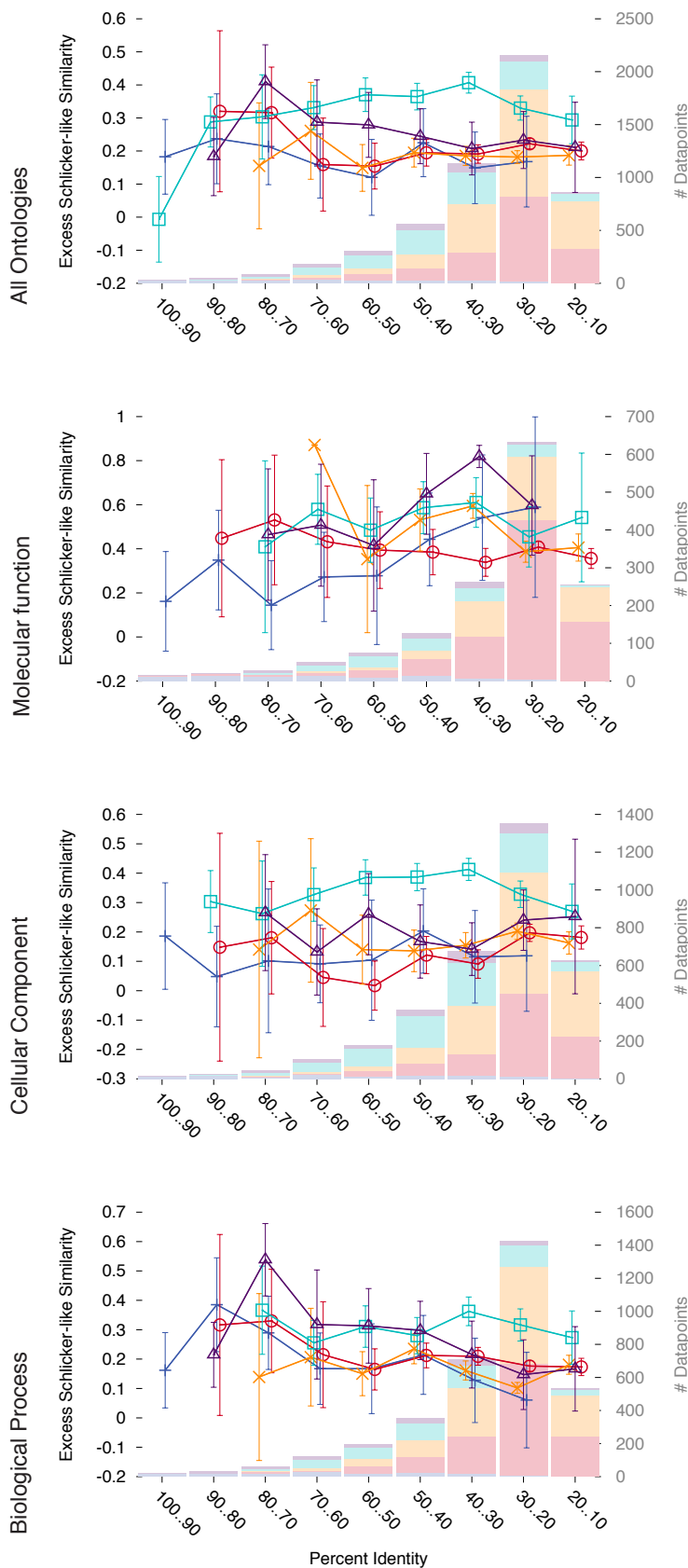

## B: PAM

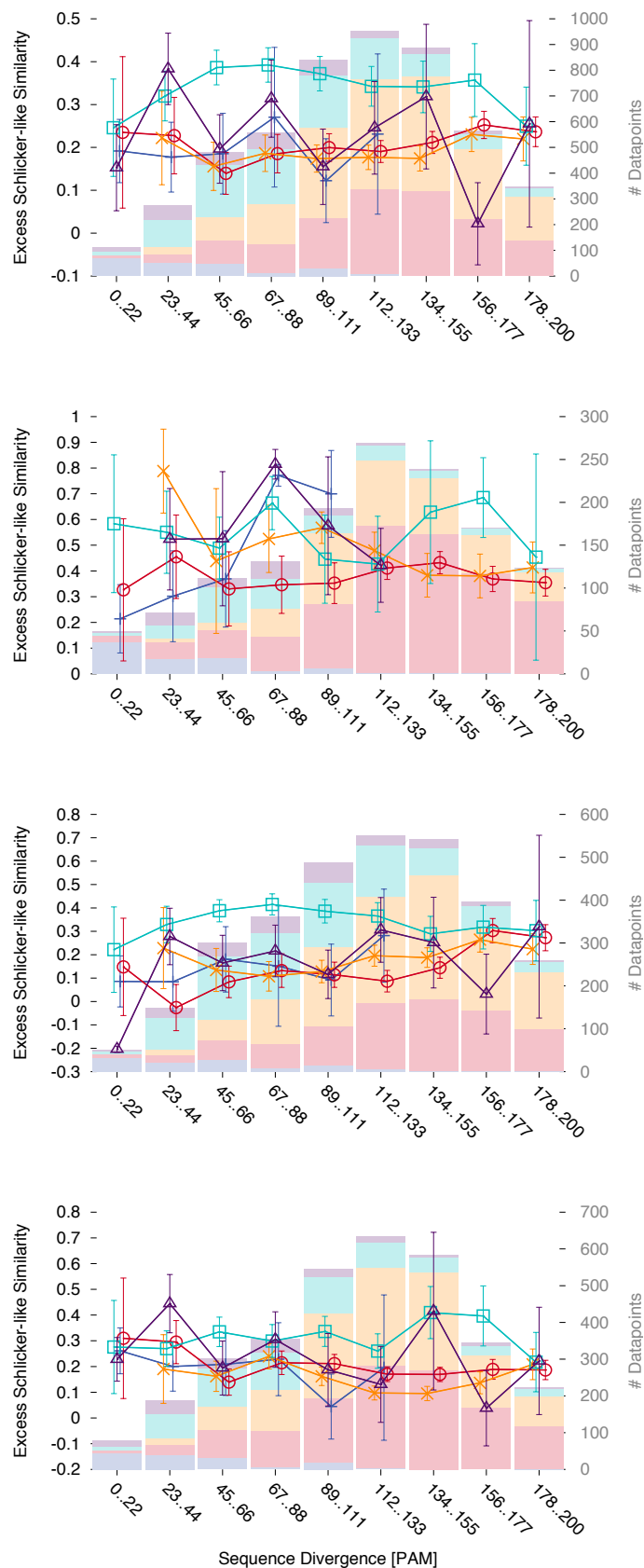

Supplement: Figure S4 — Contrasting different measures of divergence as independent variables: A) Percent sequence identity and B) PAM estimates of sequence divergence, both derived from a Smith-Waterman alignment over the full protein lengths. All function similarities are in Excess Schlicker-like Similarity and have been measured from the dataset with only GO annotations backed by experimental evidence originating from publications sharing no common authors. (PDF) [file pcbi.1002514.s005.pdf]

# Quartet Analysis

## A: Yeasts only

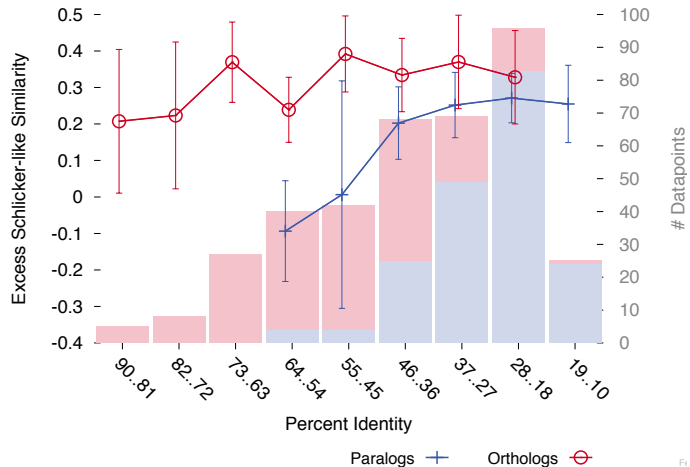

## B: All Species Pairs

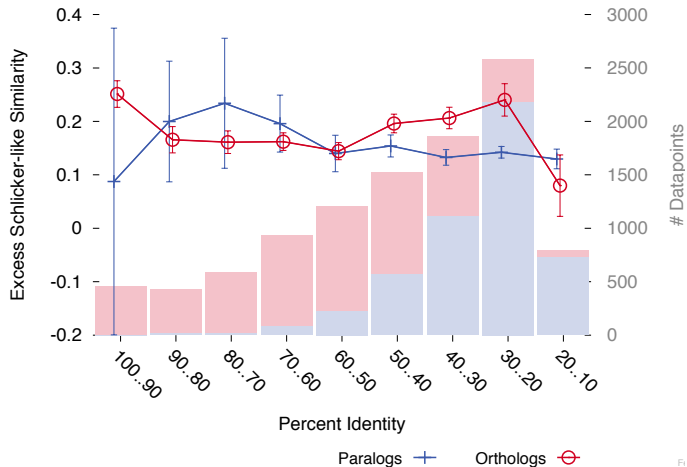

Supplement: Figure S5 — Average excess Schlicker-like Similarity measured from homologous gene pairs with GO annotations backed by experimental evidence from publications with no common authors. The sampled gene pairs form quartets with an ancient duplication and subsequent speciations. The quartets are sampled from A) the two yeast species only and B) from all 13 analyzed species. (PDF) [file pcbi.1002514.s006.pdf]

# Difference in Excess Function between Orthologs and Paralogs (Yeasts only)

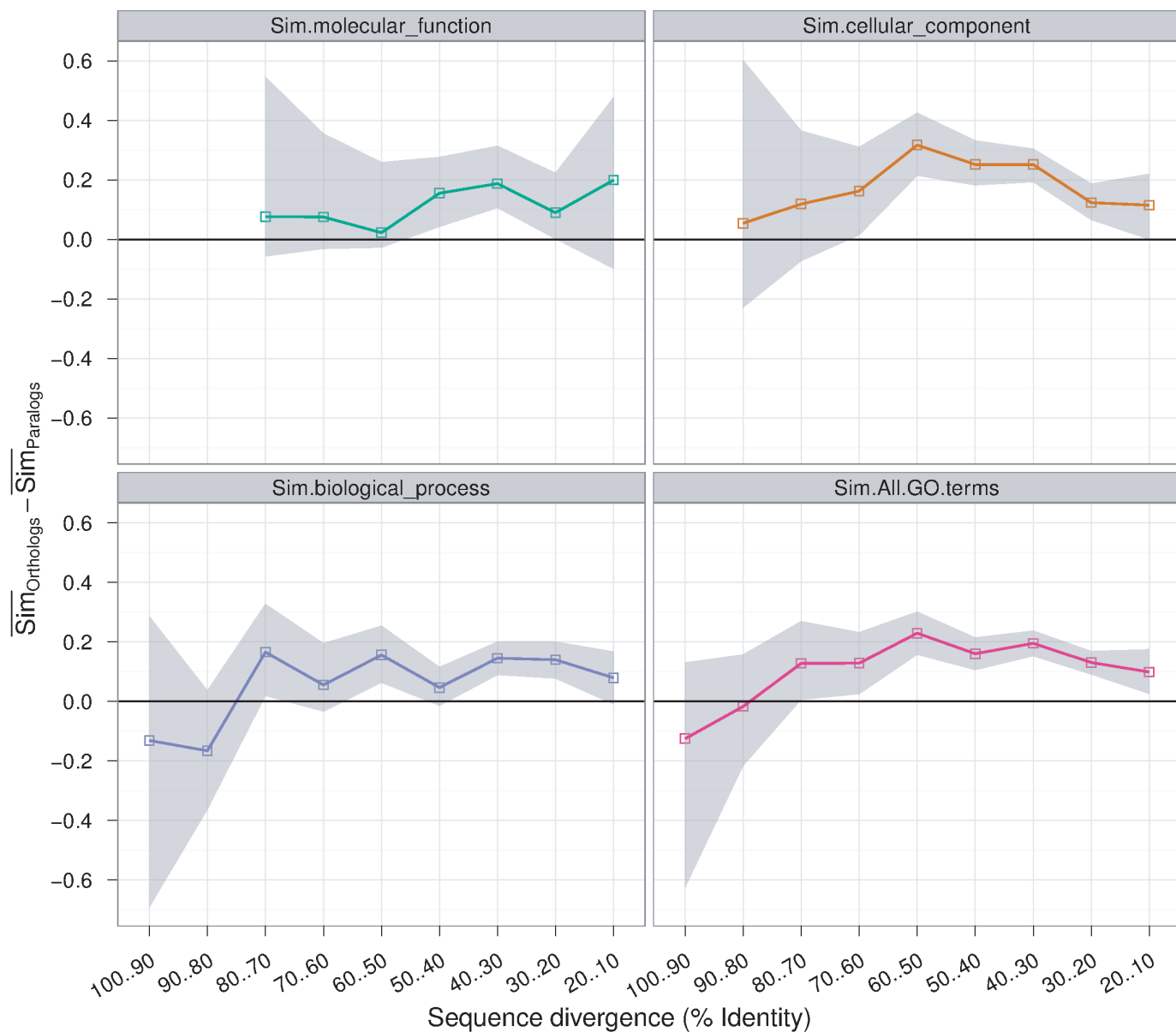

Supplement: Figure S6 — Difference in average Excess Schlicker Function Similarity between all types of Orthologs and all types of Paralogs from the YEAST/SCHPO genome pair on the dataset of pairs being backed with experimental annotations from studies without common authors. The different panels report the difference for the different GO ontologies. The data-points indicate the difference of the means and the gray area a linear interpolation of the bin-wise 95% confidence interval for the difference for the mean. To confidence interval is computed for each bin with a Mann-Whitney test. P-values are provided in Table S4 for all bins. (PDF) [file pcbi.1002514.s007.pdf]

# Analysis partitioned on all Species Pairs

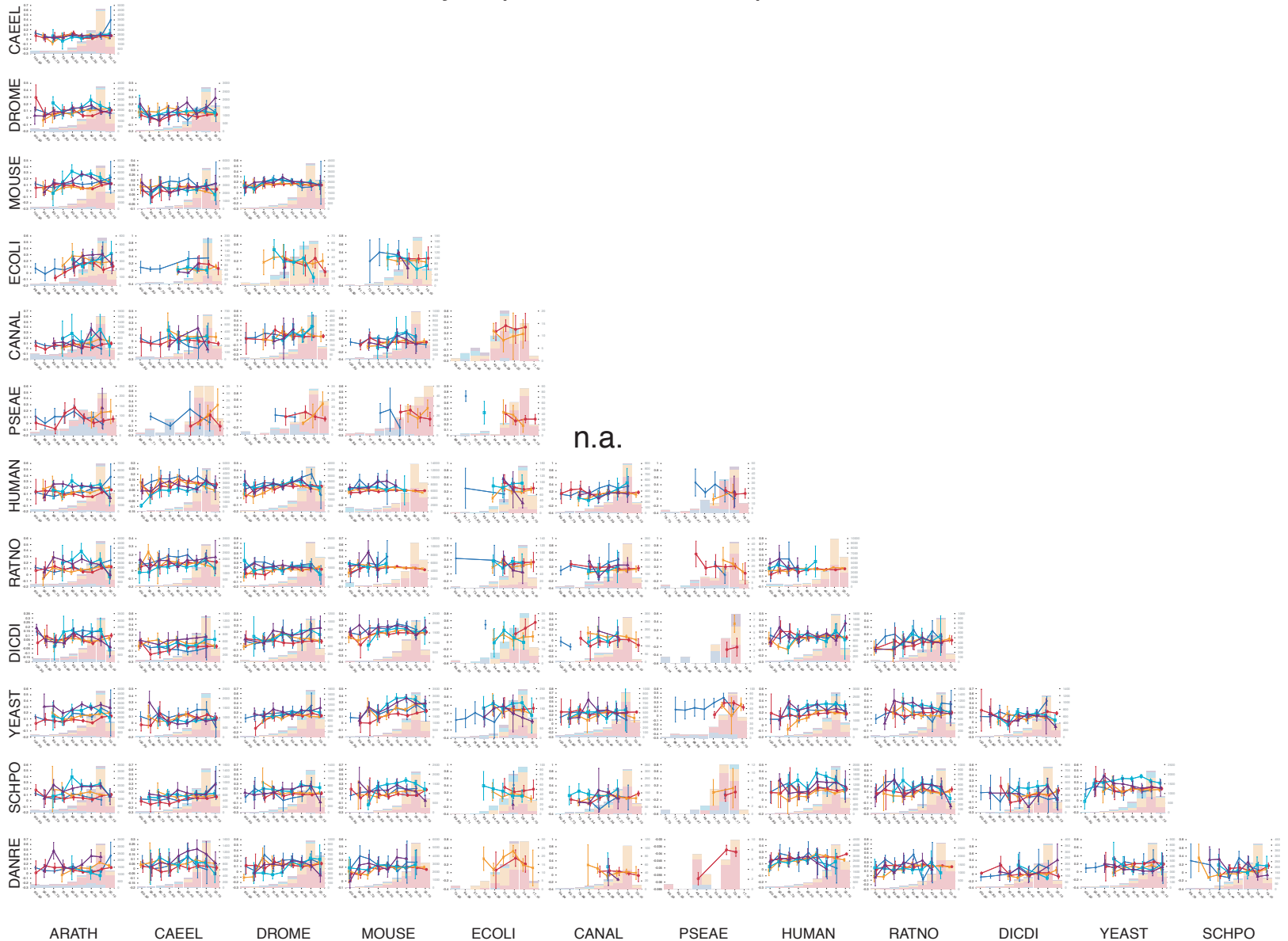

Supplement: Figure S7 — Average excess Schlicker-like similarity for any pair of analyzed species, measured on the dataset restricted to experimental annotations from publications without common authors. Reported is the average excess similarity over all three GO ontologies. A mapping of the species abbreviations to scientific names is provided in Table S3. (PDF) [file pcbi.1002514.s008.pdf]

# Difference in Excess Function between Orthologs and Paralogs

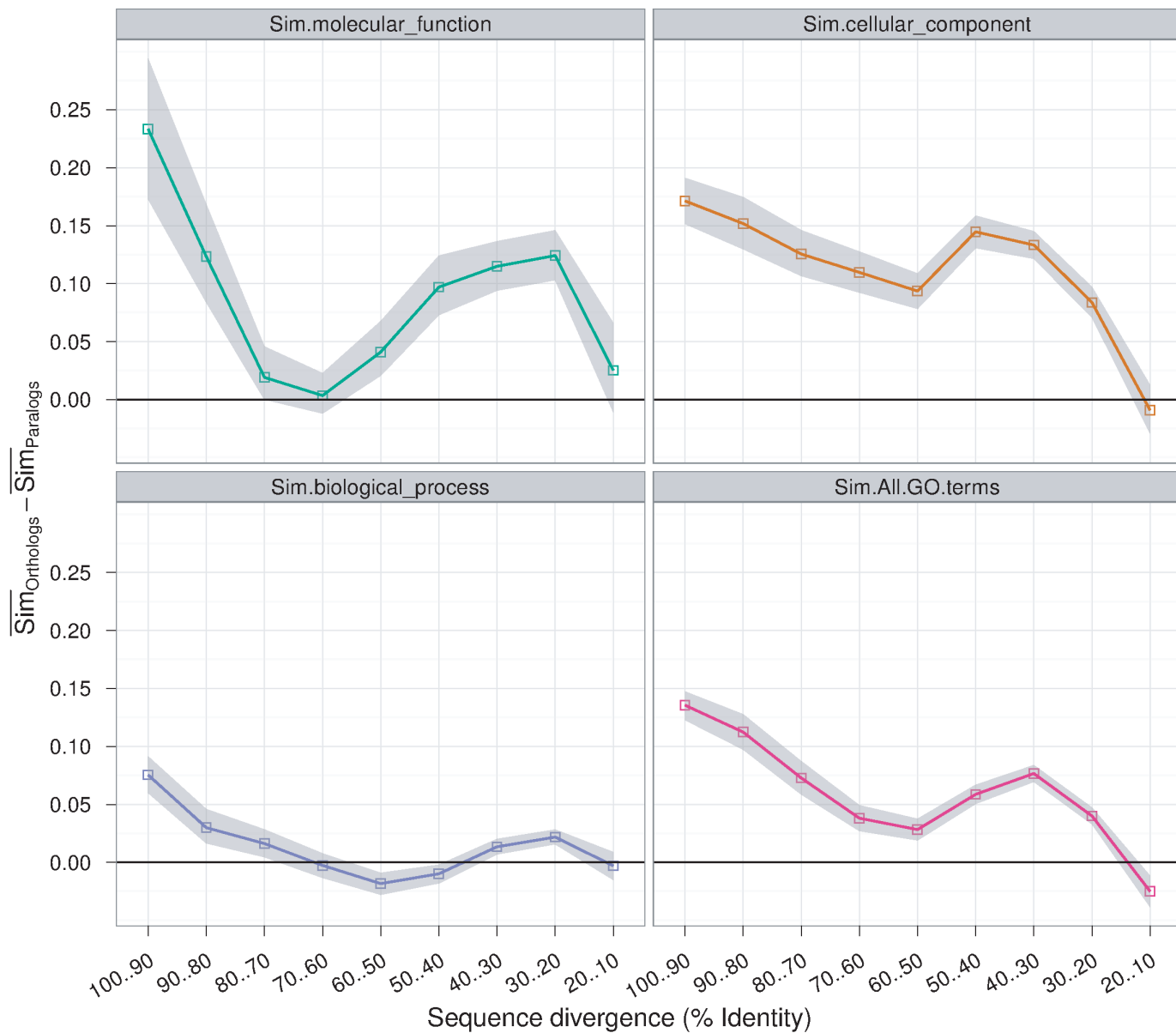

Supplement: Figure S8 — Difference in average Excess Schlicker Function Similarity between all types of Orthologs and all types of Paralogs from all 13 analyzed genomes on the dataset of pairs being backed with experimental annotations from studies without common authors. The different panels report the difference for the different GO ontologies. The data-points indicate the difference of the means and the gray area a linear interpolation of the bin-wise 95% confidence interval for the difference for the mean. To confidence interval is computed for each bin with a Mann-Whitney test. P-values for the statistical test whether the difference is different from 0 are available in Table S4 for each distance bin. (PDF) [file pcbi.1002514.s009.pdf]

## Control for domination of single species pair

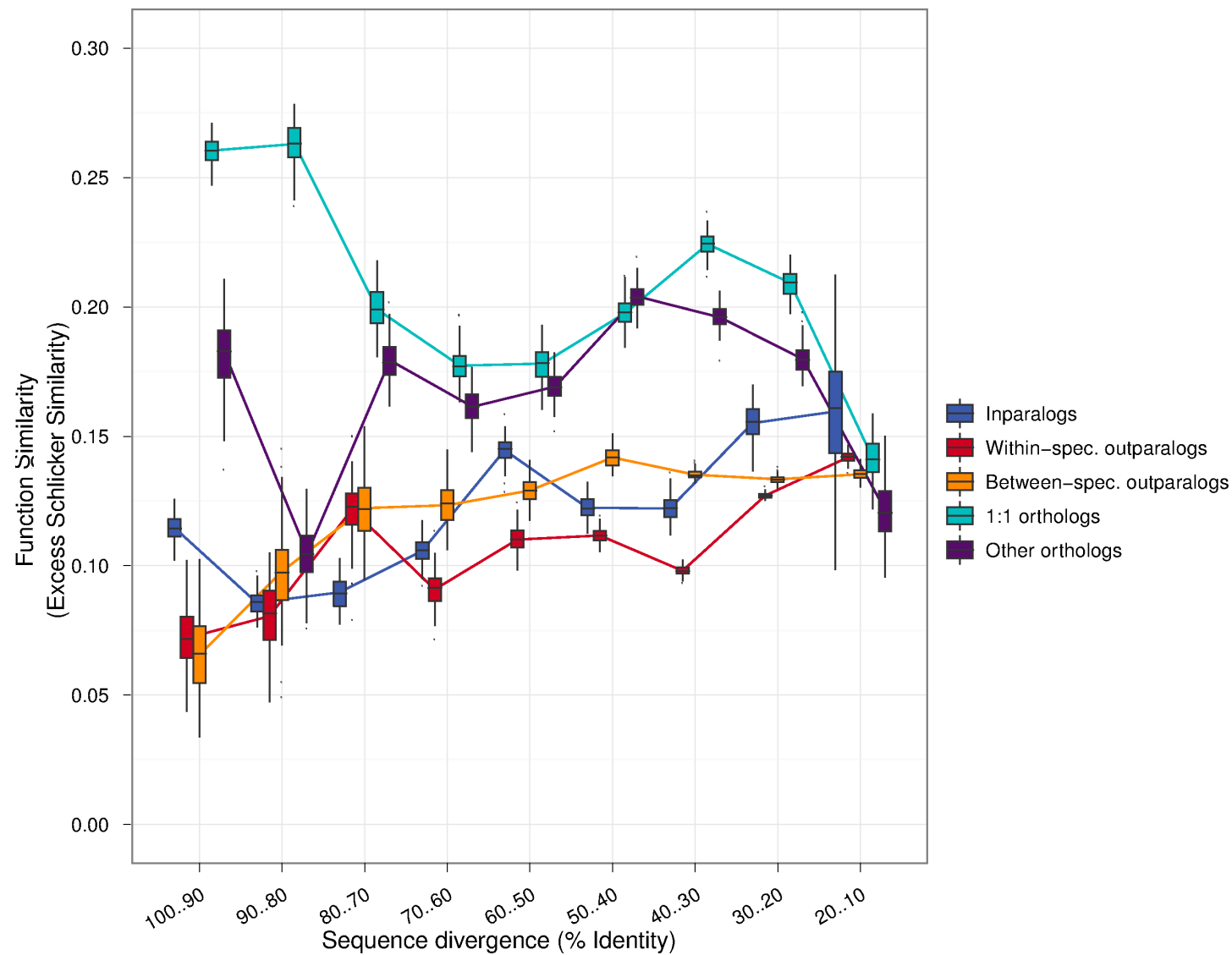

Supplement: Figure S11 — Test of over-representation of a single species pair. We applied the following re-sampling strategy to the dataset of gene pairs with experimental GO annotations without common authors: First, we partition the dataset into independent sub-datasets. Each sub-dataset is composed of all the gene pairs of a given homology type and species pair. After building those sub-datasets, we randomly select gene pairs with replacement of the same size or a maximum number of allowed pairs. This number has been set to 2000 gene pairs per species pair and homology type. This way we ensure that any species pair can influence the results more than 1.5%. We then compute the average similarity per homology type and distance category from the combined sub-datasets. This whole procedure is repeated 100 times in order to obtain the necessary quantiles for the box-plots. (PDF) [file pcbi.1002514.s012.pdf]

# Control for gene family over-representation (OMA homologs)

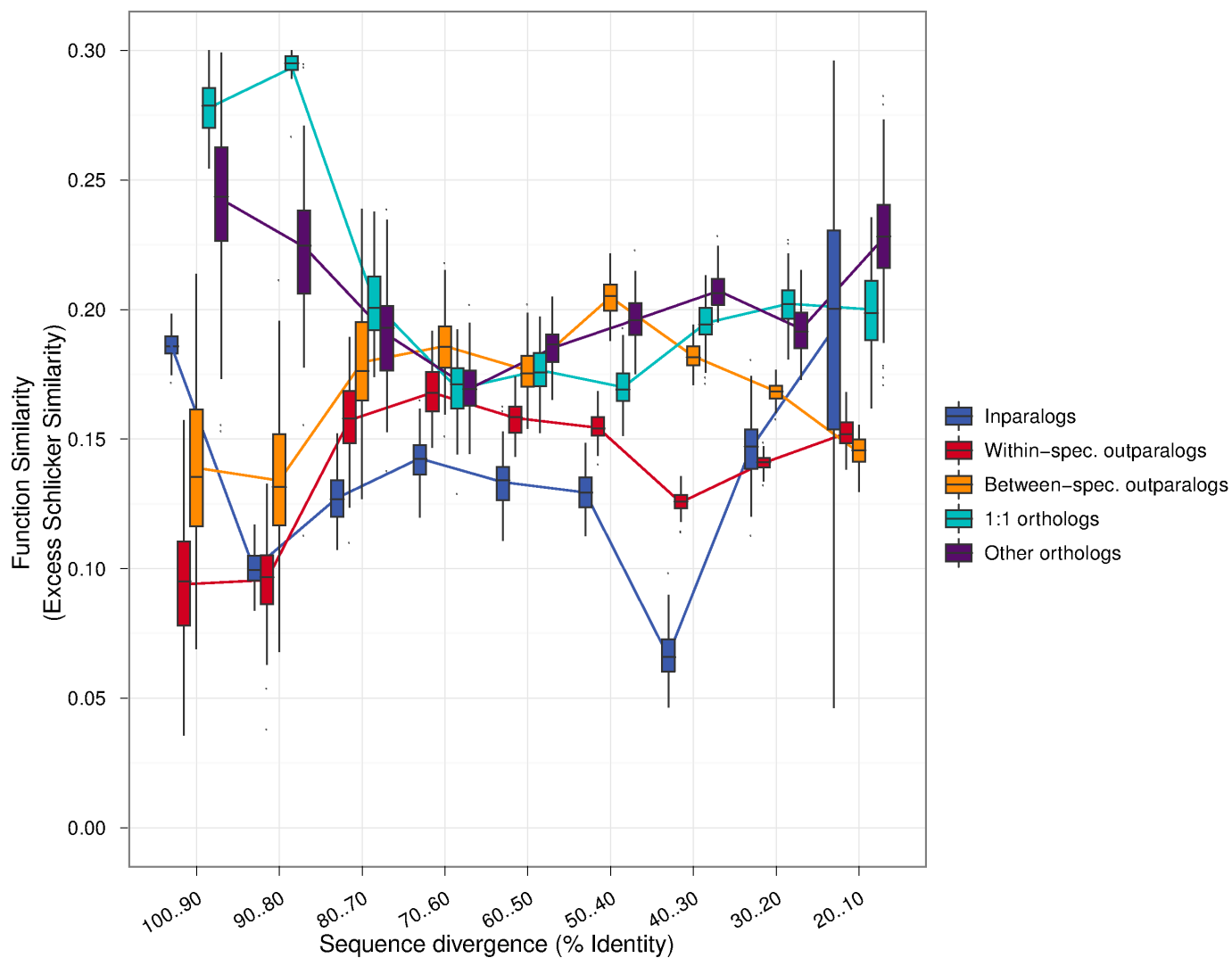

Supplement: Figure S12 — Test for over-representation of large gene families in the OMA homologs. We applied the following re-sampling strategy to the dataset of gene pairs with experimental GO annotations without common authors: First, we partition the dataset into independent sub-datasets. Each sub-dataset is composed of all the gene pairs from a given gene family. After building those sub-datasets, we randomly select gene pairs with replacement of the same size or a maximum number of allowed pairs. This number has been set to 100 gene pairs per gene family. This way we ensure that any single family can influence the results more than 1%. We then compute the average similarity per homology type and distance category from the combined sub-datasets. This whole procedure is repeated 100 times in order to obtain the necessary quantiles for the box-plots. For every gene family, we sample at most 100 homologous gene pairs with replacement. Shown are box-plots for all 100 bootstrap samples. (PDF) [file pcbi.1002514.s013.pdf]

# Enzyme Commission numbers

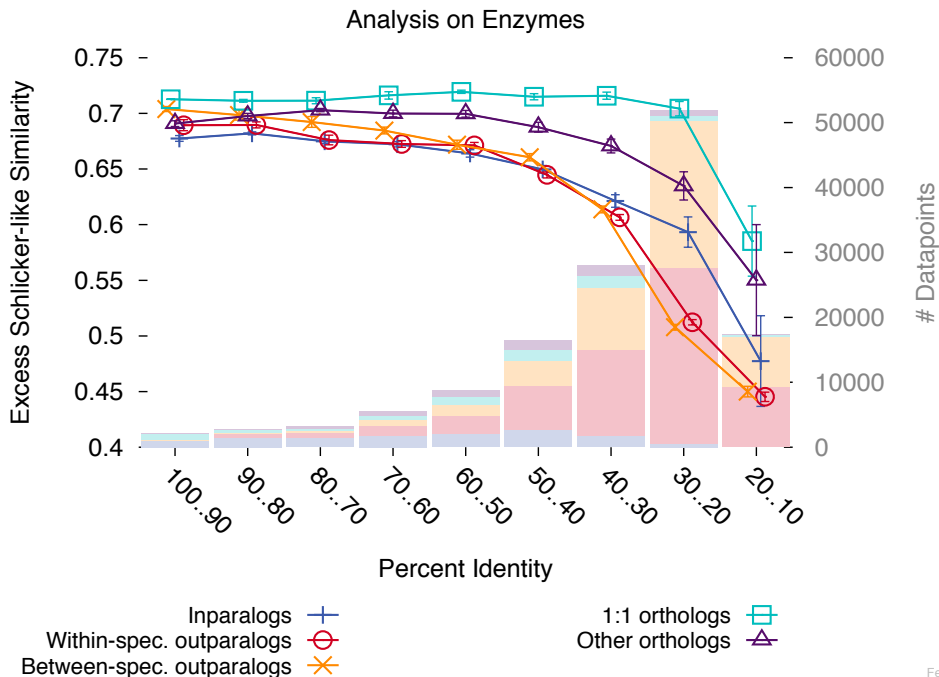

Supplement: Figure S15 — Average excess Schlicker-like similarity of the various types of homologs with EC number annotations, with sequence divergence in percent identity as independent variable. (PDF) [file pcbi.1002514.s016.pdf]

# Additional analysis of data from Nehrt *et al*

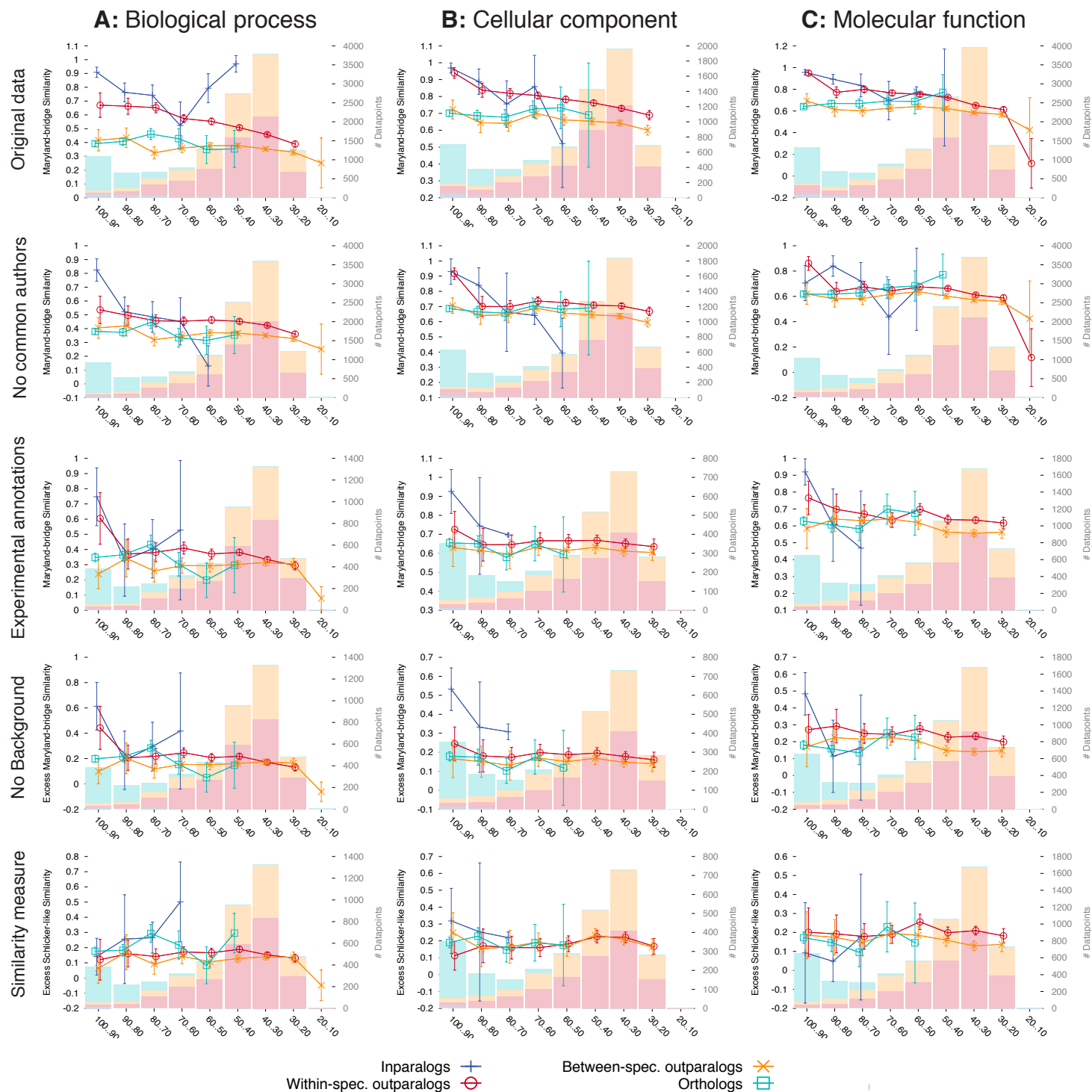

Supplement: Figure S16 — Effect sequence on functional similarity after correcting for several biases for A) biological process, B) cellular component and C) molecular function GO ontology. Homologs are taken from Nehrt et. al (2011), and initial plots are computed on experimental GO annotations augmented with curated annotations having TAS or IC evidence code. In the subsequent plots, we correct for author bias (only annotations from publications without common author), curator effect (by only looking at experimental annotations), varying background and information content based similarity measure. (PDF) [file pcbi.1002514.s017.pdf]

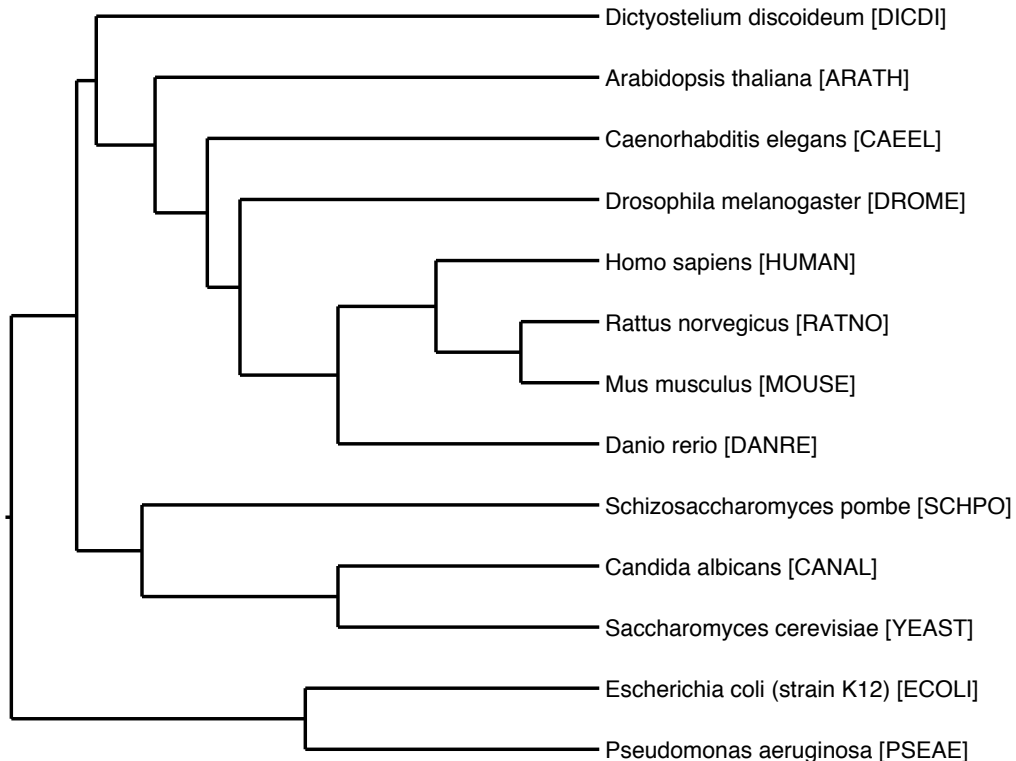

Supplement: Figure S17 — The 13 species used in the analysis and their phylogenetic relations among each other according to the NCBI taxonomy. (PDF) [file pcbi.1002514.s018.pdf]
