# Supplementary material for: Resolving the Ortholog Conjecture: Orthologs Tend to Be Weakly, but Significantly, More Similar in Function than Paralogs
Source: PLoS Comput Biol. 2012 May 17;8(5):e1002514. doi: 10.1371/journal.pcbi.1002514 (PMC3355068; doi:10.1371/journal.pcbi.1002514)

Influence of Bin-width

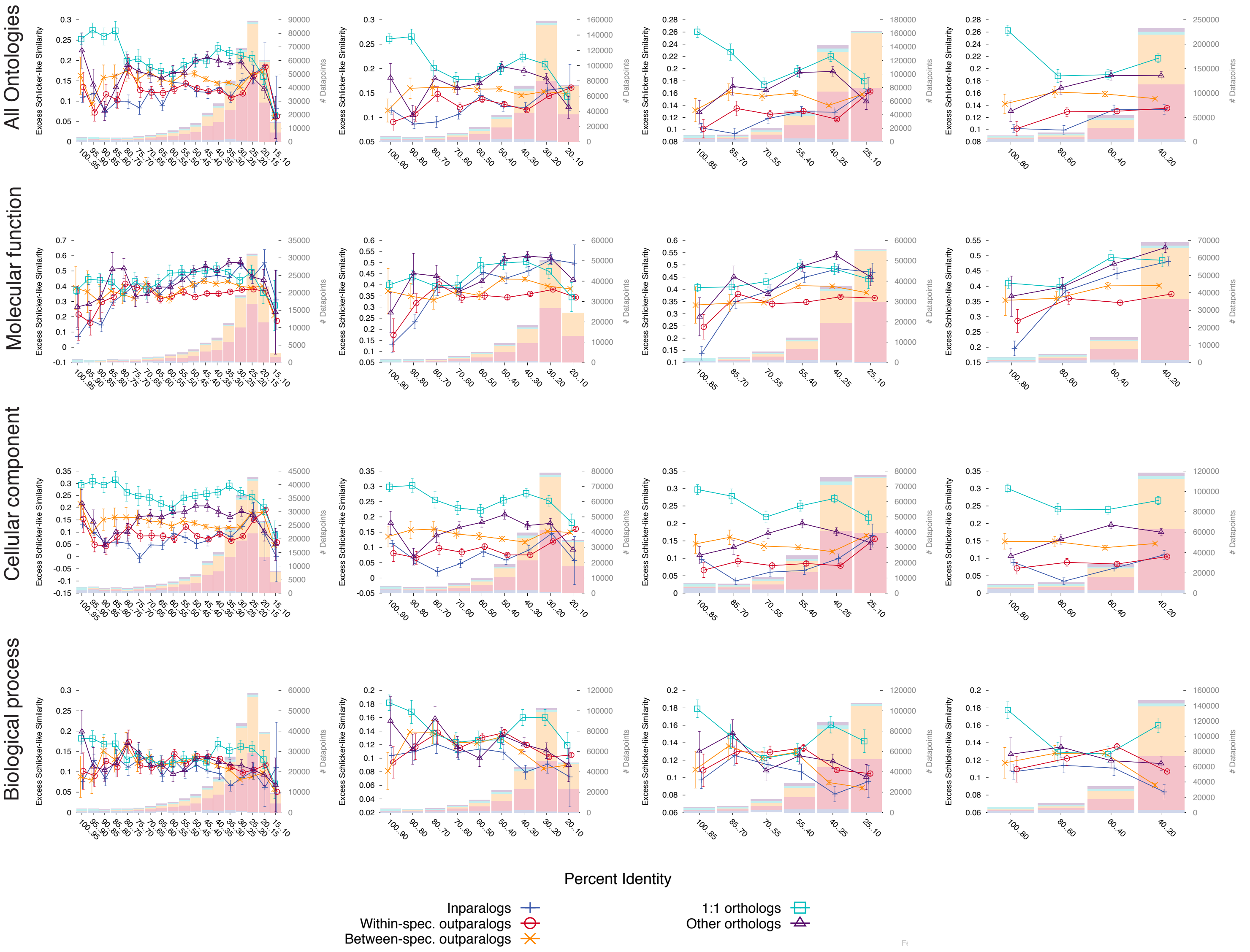

Supplement: Figure S9 — Different bin-widths (columns) for evolutionary divergence categories: the results are robust with respect to the choice of bin width. The analysis is done on the gene pairs with experimental GO annotations without common author between all 13 genomes. (PDF) [file pcbi.1002514.s010.pdf]
