# Supplementary material for: Resolving the Ortholog Conjecture: Orthologs Tend to Be Weakly, but Significantly, More Similar in Function than Paralogs
Source: PLoS Comput Biol. 2012 May 17;8(5):e1002514. doi: 10.1371/journal.pcbi.1002514 (PMC3355068; doi:10.1371/journal.pcbi.1002514)
Supplement: Figure S10 — Different measures of GO term similarity among various types of homologs. The six figures are A) maximum simResnik, B) average simResnik, C) maximum simLin and D) average simLin, E) Maryland-bridge term overlap measure, F) simSchlicker (giving same weight to annotation) and G) simSchlicker as originally defined in Schlicker et. al (2006) (giving same weight to each gene product). All similarities are measured from the gene pair s from all 13 analyzed genomes with GO annotations backed by experimental evidence without common authors. (PDF) [file pcbi.1002514.s011.pdf]

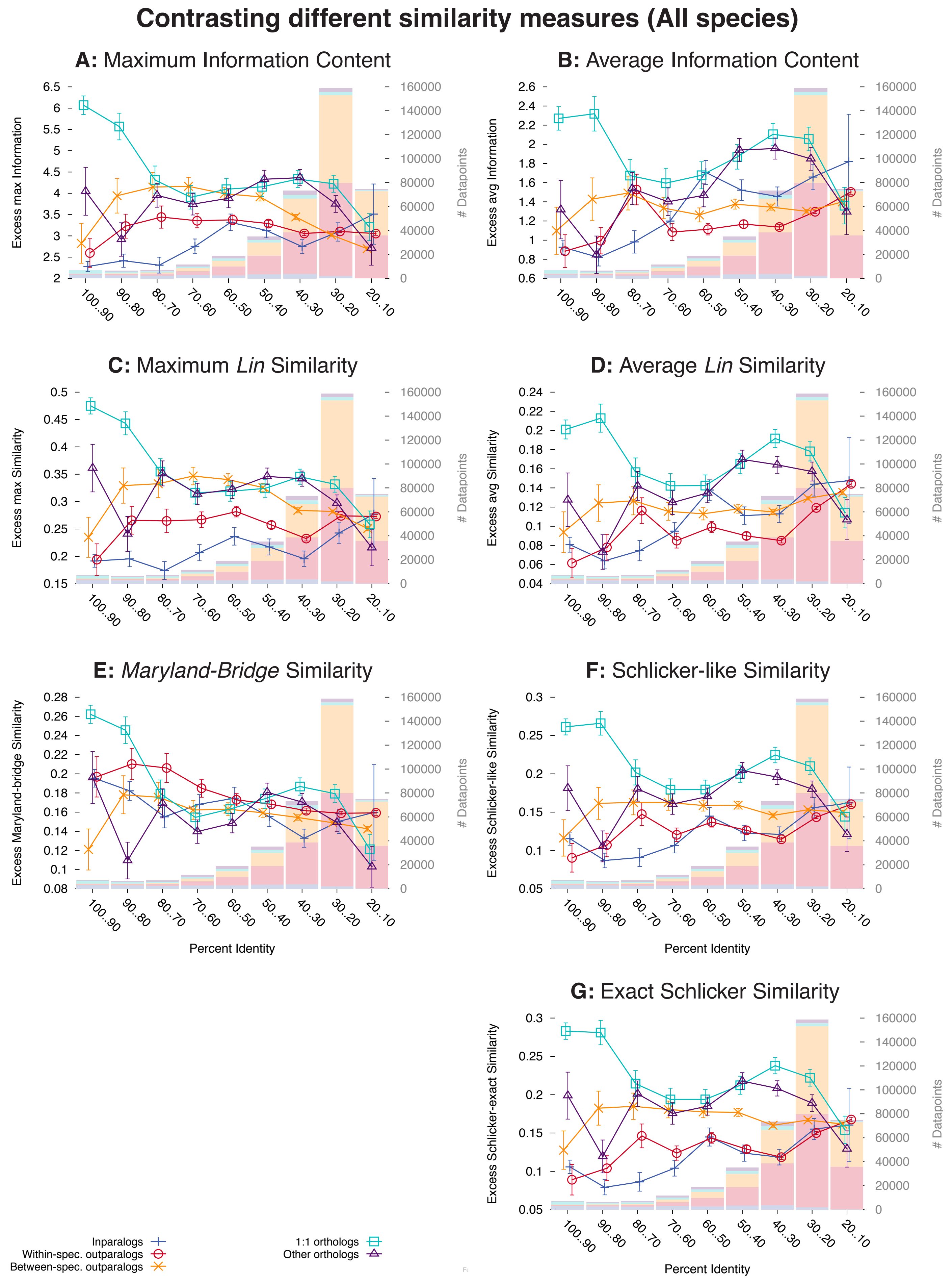
Contrasting different similarity measures (All species)
A: Maximum Information Content
Excess max Information
B: Average Information Content
Excess avg Information
C: Maximum Lin Similarity
Excess max Similarity
D: Average Lin Similarity
Excess avg Similarity
E: Maryland-Bridge Similarity
Excess Maryland-bridge Similarity
F: Schlicker-like Similarity
Excess Schlicker-like Similarity
G: Exact Schlicker Similarity
Excess Schlicker-exact Similarity
# Datapoints
100..90
90..80
80..70
70..60
60..50
50..40
40..30
30..20
20..10
Percent Identity
Inparalogs
Within-spec. outparalogs
Between-spec. outparalogs
1:1 orthologs
Other orthologs
