# Supplementary material for: Resolving the Ortholog Conjecture: Orthologs Tend to Be Weakly, but Significantly, More Similar in Function than Paralogs
Source: PLoS Comput Biol. 2012 May 17;8(5):e1002514. doi: 10.1371/journal.pcbi.1002514 (PMC3355068; doi:10.1371/journal.pcbi.1002514)
Supplement: Figure S13 — Orthology/Paralogy relations inferred from Ensembl Gene Trees (version 65). To control for a potential bias in the orthology/paralogy inference method we repeated the analysis on homologs induced by the labeled Ensembl gene trees. Note that this analysis is limited to the following 6 species: HUMAN, MOUSE, RATNO, DROME, CAEEL and YEAST. Shown are the excess Schlicker similarities. In all ontologies, orthologs are significantly more similar in function than paralogs. The figures show the similarities of A) the average over all gene ontologies (t-test: p<2.2E−16), B) the molecular function ontology (t-test: p<2.2E−16), C) the biological process ontology (t-test: p = 2.19E−6) and D) the cellular component ontology (t-test: p<2.2E−16). All similarities have been computed on the dataset with experimental annotations without common authors from GOA 2012-01-21. (PDF) [file pcbi.1002514.s014.pdf]

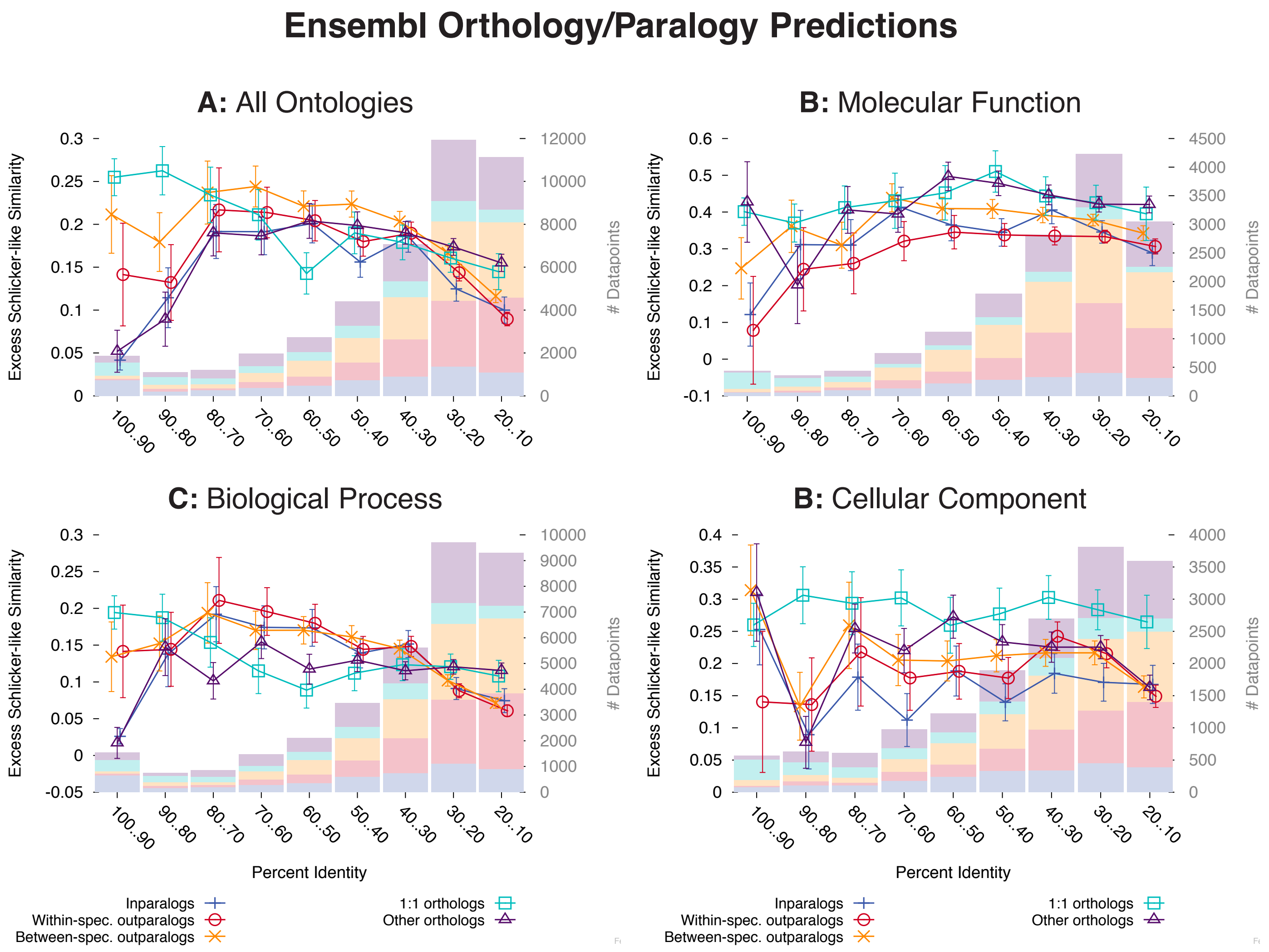
Ensembl Orthology/Paralogy Predictions
A: All Ontologies
B: Molecular Function
C: Biological Process
B: Cellular Component
Excess Schlicker-like Similarity
Datapoints
Percent Identity
100..90
90..80
80..70
70..60
60..50
50..40
40..30
30..20
20..10
Inparalogs
Within-spec. outparalogs
Between-spec. outparalogs
1:1 orthologs
Other orthologs
