# Supplementary material for: Resolving the Ortholog Conjecture: Orthologs Tend to Be Weakly, but Significantly, More Similar in Function than Paralogs
Source: PLoS Comput Biol. 2012 May 17;8(5):e1002514. doi: 10.1371/journal.pcbi.1002514 (PMC3355068; doi:10.1371/journal.pcbi.1002514)

Different metrics of protein divergence

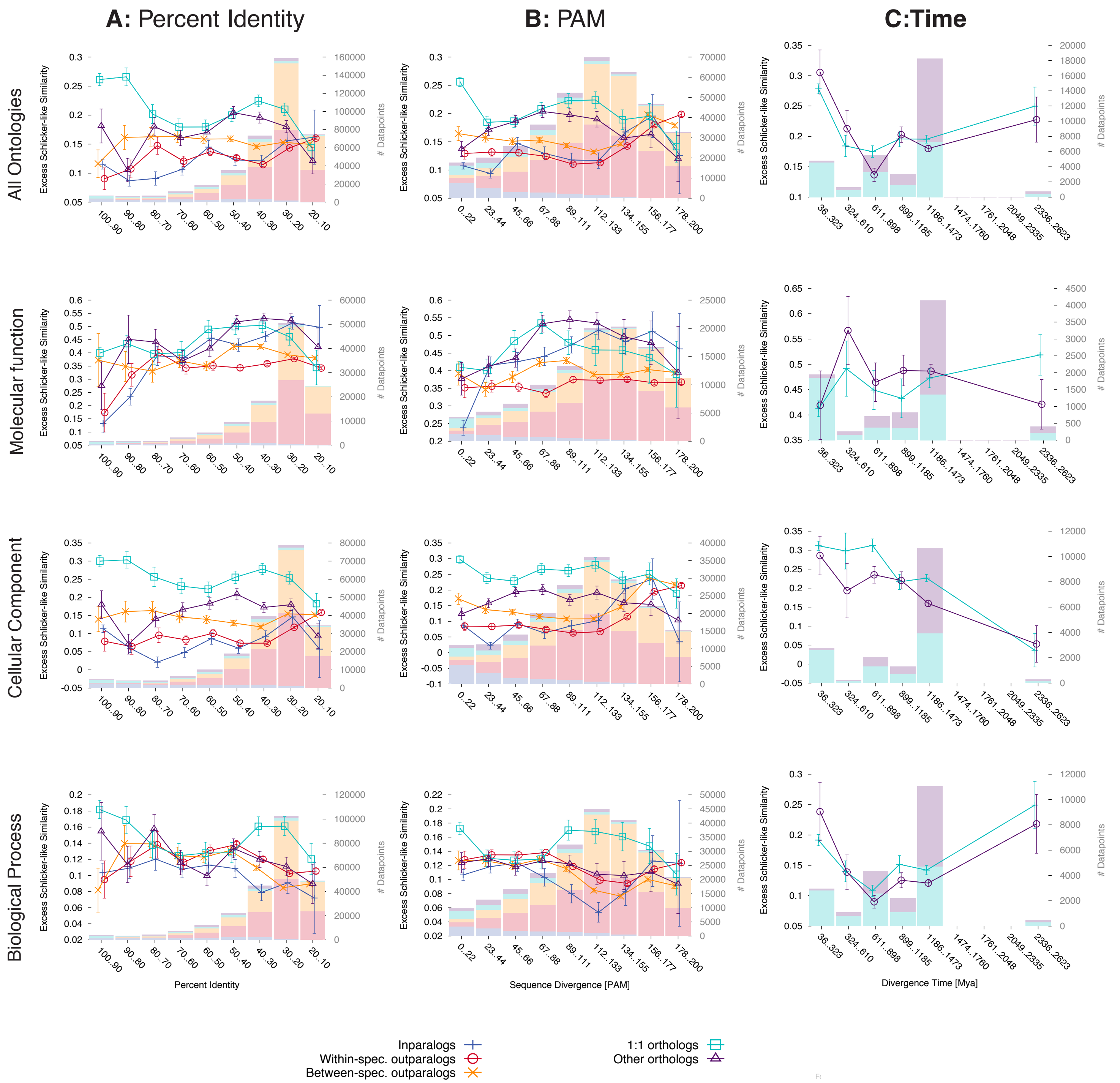

Supplement: Figure S14 — Contrasting different measures of divergence as independent variables: A) Percent sequence identity, B) PAM estimates of sequence divergence and C) Time estimates. Time estimates have been extracted from TimeTree (http://timetree.org). All function similarities are in Excess Schlicker-like Similarity and have been measured from the dataset with only GO annotations backed by experimental evidence originating from publications sharing no common authors. (PDF) [file pcbi.1002514.s015.pdf]
