# Supplementary material for: Resolving the Ortholog Conjecture: Orthologs Tend to Be Weakly, but Significantly, More Similar in Function than Paralogs
Source: PLoS Comput Biol. 2012 May 17;8(5):e1002514. doi: 10.1371/journal.pcbi.1002514 (PMC3355068; doi:10.1371/journal.pcbi.1002514)
Supplement: Table S1 — Authorship bias: the fraction of homologs with experimental GO annotations from the same publication, different publication but common author and different authors varies strongly. All homologs have at least 50% sequence identity. (PDF) [file pcbi.1002514.s019.pdf]

	Same Paper		Different Paper, same Author		Different Authors	
Inparalogs	14090	(58.70%)	3956	(40.31%)	14586	(28.25%)
Within-spec. outparalogs	9288	(38.70%)	3676	(37.46%)	13481	(26.11%)
Between-spec. outparalogs	88	(0.37%)	604	(6.15%)	9045	(17.52%)
1:1 orthologs	505	(2.10%)	1350	(13.76%)	8696	(16.84%)
Other orthologs	32	(0.13%)	228	(2.32%)	5831	(11.29%)
Avg Similarity	0.7086 ± 0.0023		0.2377 ± 0.0068		0.1499 ± 0.0024	
