# Supplementary material for: Resolving the Ortholog Conjecture: Orthologs Tend to Be Weakly, but Significantly, More Similar in Function than Paralogs
Source: PLoS Comput Biol. 2012 May 17;8(5):e1002514. doi: 10.1371/journal.pcbi.1002514 (PMC3355068; doi:10.1371/journal.pcbi.1002514)
Supplement: Table S2 — Authorship bias: equivalent to Table S1, but without restriction on the sequence conservation. (PDF) [file pcbi.1002514.s020.pdf]

	Same Paper		Different Paper, same Author		Different Authors	
Inparalogs	19722	(20.31%)	6378	(15.33%)	23836	(5.99%)
Within-spec. outparalogs	76446	(78.73%)	28891	(69.45%)	179289	(45.03%)
Between-spec. outparalogs	266	(0.27%)	3767	(9.05%)	160899	(40.41%)
1:1 orthologs	602	(0.62%)	1940	(4.66%)	18341	(4.60%)
Other orthologs	60	(0.06%)	622	(1.50%)	15789	(3.97%)
Avg Similarity	0.6942 ± 0.0013		0.2345 ± 0.0033		0.1477 ± 0.0008	
