# Supplementary material for: Resolving the Ortholog Conjecture: Orthologs Tend to Be Weakly, but Significantly, More Similar in Function than Paralogs
Source: PLoS Comput Biol. 2012 May 17;8(5):e1002514. doi: 10.1371/journal.pcbi.1002514 (PMC3355068; doi:10.1371/journal.pcbi.1002514)
Supplement: Table S3 — Significance test for difference of mean excess Schlicker-like similarity between orthologs and paralogs. P-values have been computed for each distance bin separately using a Mann-Whitney test. Values are shown for the dataset covering all 13 genomes (middle column) as well as the yeast-only dataset (rightmost column). The corresponding graphs are provided in Fig. S6 (yeast only) and S8 (all species). (PDF) [file pcbi.1002514.s021.pdf]

		All Species		Yeasts only	
Ontology	Distance	Sim Difference	p-value	Sim Difference	p-value
Molecular Function	100..90	0.2334	7.5360e-47	n/a	n/a
Molecular Function	90..80	0.1233	2.2688e-19	n/a	n/a
Molecular Function	80..70	0.0191	0.04995	0.0769	0.22049
Molecular Function	70..60	0.0034	0.66286	0.0756	0.14903
Molecular Function	60..50	0.0410	2.0659e-06	0.0228	0.14823
Molecular Function	50..40	0.0970	9.6769e-25	0.1555	0.00079
Molecular Function	40..30	0.1149	1.6336e-42	0.1878	3.6373e-08
Molecular Function	30..20	0.1242	7.3748e-39	0.0902	0.04669
Molecular Function	20..10	0.0252	0.18508	0.1996	0.17939
Cellular Component	100..90	0.1713	8.5209e-74	n/a	n/a
Cellular Component	90..80	0.1518	7.4402e-45	0.0544	0.44085
Cellular Component	80..70	0.1255	4.4436e-37	0.1192	0.27787
Cellular Component	70..60	0.1097	1.4986e-39	0.1627	0.01967
Cellular Component	60..50	0.0935	3.1506e-37	0.3172	7.2670e-08
Cellular Component	50..40	0.1447	4.0671e-103	0.2519	1.1496e-09
Cellular Component	40..30	0.1333	3.4078e-118	0.2519	3.4002e-16
Cellular Component	30..20	0.0837	4.7042e-40	0.1238	5.5011e-06
Cellular Component	20..10	-0.0091	0.41042	0.1152	0.05008
Biological Process	100..90	0.0753	8.1355e-26	-0.1314	0.58919
Biological Process	90..80	0.0298	7.2031e-06	-0.1659	0.11915
Biological Process	80..70	0.0161	0.00836	0.1657	0.03169
Biological Process	70..60	-0.0026	0.61199	0.0554	0.30759
Biological Process	60..50	-0.0183	8.9048e-05	0.1560	0.00010
Biological Process	50..40	-0.0100	0.01142	0.0466	0.15033
Biological Process	40..30	0.0133	5.9868e-05	0.1454	1.7766e-08
Biological Process	30..20	0.0217	4.4363e-11	0.1399	1.2702e-07
Biological Process	20..10	-0.0031	0.58269	0.0791	0.08826
All Ontologies	100..90	0.1355	8.1082e-91	-0.1253	0.39706
All Ontologies	90..80	0.1123	1.6987e-48	-0.0170	0.89705
All Ontologies	80..70	0.0727	1.5308e-23	0.1279	0.03892
All Ontologies	70..60	0.0380	1.8090e-11	0.1285	0.01085
All Ontologies	60..50	0.0282	2.8048e-09	0.2293	1.7291e-09
All Ontologies	50..40	0.0586	2.0193e-45	0.1603	1.2732e-08
All Ontologies	40..30	0.0765	5.3908e-102	0.1951	8.0074e-20
All Ontologies	30..20	0.0399	4.6771e-28	0.1307	1.4446e-10
All Ontologies	20..10	-0.0250	0.00022	0.0988	0.00923
