# Supplementary material for: Resolving the Ortholog Conjecture: Orthologs Tend to Be Weakly, but Significantly, More Similar in Function than Paralogs
Source: PLoS Comput Biol. 2012 May 17;8(5):e1002514. doi: 10.1371/journal.pcbi.1002514 (PMC3355068; doi:10.1371/journal.pcbi.1002514)
Supplement: Table S4 — Species information: source and release date for all 13 analyzed species. Their phylogenetic relation is depicted in Fig. S17. (PDF) [file pcbi.1002514.s022.pdf]

Scientific Species Name	Species-code	NCBI Taxon ID	DB source	DB release
Arabidopsis thaliana	ARATH	3702	NCBI	NC_003070.5; GI:42592260; 04-NOV-2005
Caenorhabditis elegans	CAEEL	6239	Ensembl	Ensembl 46; WS170; 4-AUG-2007
Candida albicans	CANAL	5476	EBI genomes	12-JUN-2009 (Rel. 101)
Danio rerio	DANRE	7955	Ensembl	Ensembl v57; Zv8; 2-FEB-2010
Dictyostelium discoideum	DICDI	44689	dictyBase	release-2-12; 08-30-2009 01:33
Drosophila melanogaster	DROME	7227	Ensembl	Ensembl 46; BDGP 4.3; 5-AUG-2007
Escherichia coli (strain K12)	ECOLI	511145	Genome Reviews	03-MAR-2009 (Rel. 103)
Homo sapiens	HUMAN	9606	Ensembl	Ensembl 55; GRCh37; 21-JUL-2009
Mus musculus	MOUSE	10090	Ensembl	Ensembl v48; NCBI m37; 28-NOV-2007
Pseudomonas aeruginosa	PSEAE	208964	Genome Reviews	01-SEP-2009 (Rel. 110)
Rattus norvegicus	RATNO	10116	Ensembl	Ensembl 55; RGSC3.4; 16-JUN-2009
Saccharomyces cerevisiae	YEAST	4932	EnsemblGenomes	Ensembl Fungi 4; SGD EF 1; 2-MAR-2010
Schizosaccharomyces pombe	SCHPO	4896	EnsemblGenomes	Ensembl Fungi 4; GeneDB EF 1; 17-FEB-2010
